# Multi-omics insights into bacterial and fungal bioremediation of Potentially Toxic Elements (PTEs): a critical overview of their applications

**DOI:** 10.1007/s11274-026-05253-w

**Published:** 2026-09-21

**Authors:** Maria Claudia Gatto, Flora Cozzolino, Laura Vitale, Fortunato Palma Esposito, Paola Cicatiello, Donatella de Pascale, Maria Monti

**Affiliations:** 1 Department of Chemical Sciences, University of Naples Federico II, Via Cintia 21, Naples, 80126 Italy; 2 CEINGE – Biotecnologie Avanzate “Franco Salvatore”, Via Gaetano Salvatore 486, Naples, 80145 Italy; 3 Department of Life and Environmental Sciences, Marche Polytechnic University, Via Brecce Bianche, Ancona, 60131 Italy; 4 Department of Ecosustainable Marine Biotechnology, Stazione Zoologica Anton Dohrn, Via Ammiraglio Acton 55, Naples, 80133 Italy

**Keywords:** Genomics, Heavy metals, Metabolomics, Microbial bioremediation, Proteomics, Transcriptomics

## Abstract

Potentially toxic elements (PTEs) are persistent contaminants of terrestrial and aquatic ecosystems and heavy metals and metalloids represent a major environmental and health concern. Microbial bioremediation exploits the ability of bacteria, fungi, and microbial communities to modulate PTE fate through processes including biosorption, bioaccumulation, redox transformation, biomineralization, precipitation, chelation, and extracellular sequestration. This review examines the major advances of the last decade in the application of genomics, transcriptomics, proteomics, and metabolomics and their integration, to investigate molecular mechanisms of microbial adaptation to PTE contamination, supporting the selection of suitable microorganisms or microbial communities and the development of more effective bioremediation strategies. Genomic and metagenomic analyses enable the identification of genes and gene families associated with PTE resistance and adaptation, revealing both metal-specific and more general responses according to the presence of operons and/or cluster genes. Transcriptomic and proteomic approaches are applied to validate genetic potentialities, identifying mechanisms and protein mediators for transport, detoxification, redox homeostasis, and metal interactions. Metabolomics complements these approaches by characterizing metabolites involved in microbial responses, including organic acids, siderophores, biosurfactants, and extracellular polymeric substance-associated compounds. The review also discusses the advantages, limitations, and complementarity of the different omics approaches, emphasizing their impact in feasibility to move from ex situ to in situ applications. Finally, the review also addresses how omics layers could be combined across the phases of a real bioremediation project (screening, implementation, monitoring), highlighting integrated multi-omics approaches as powerful tools for developing and optimizing effective bioremediation strategies.

**Graphical abstract:**

**Figure:**
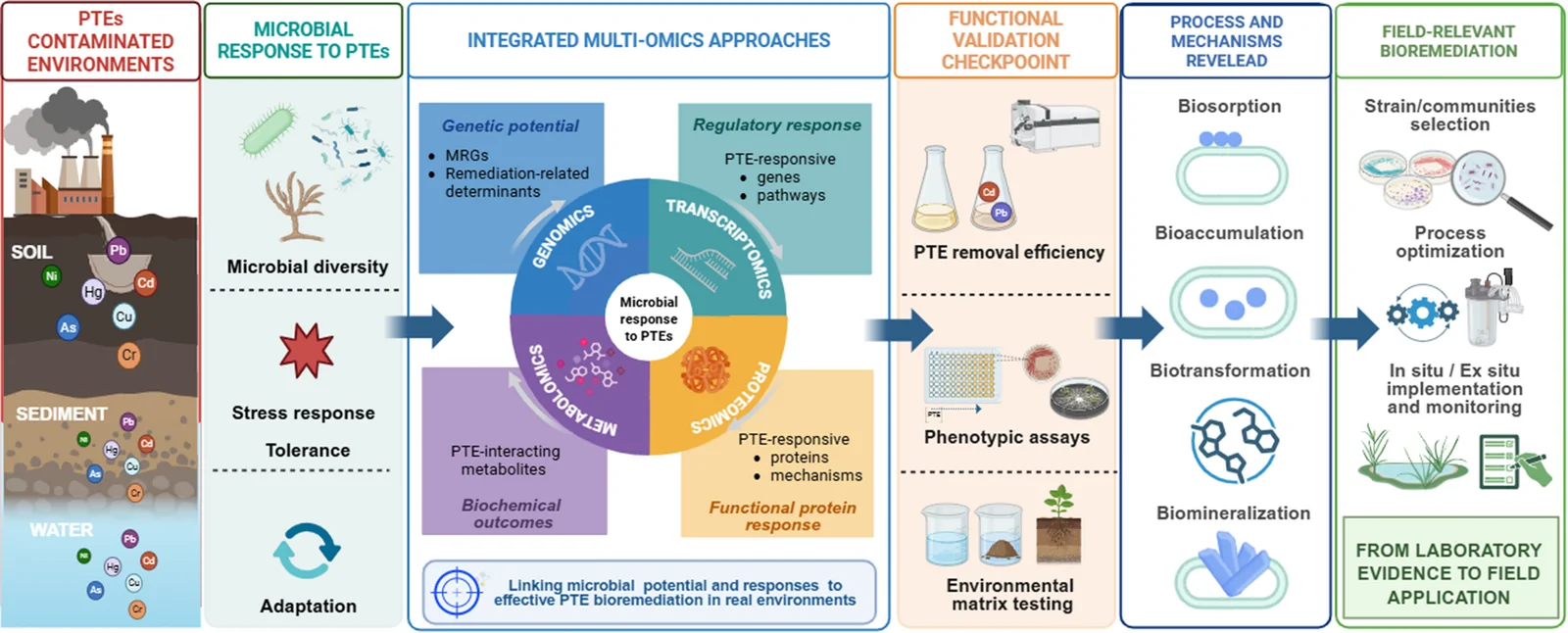

## Introduction

**Fig. 1:**
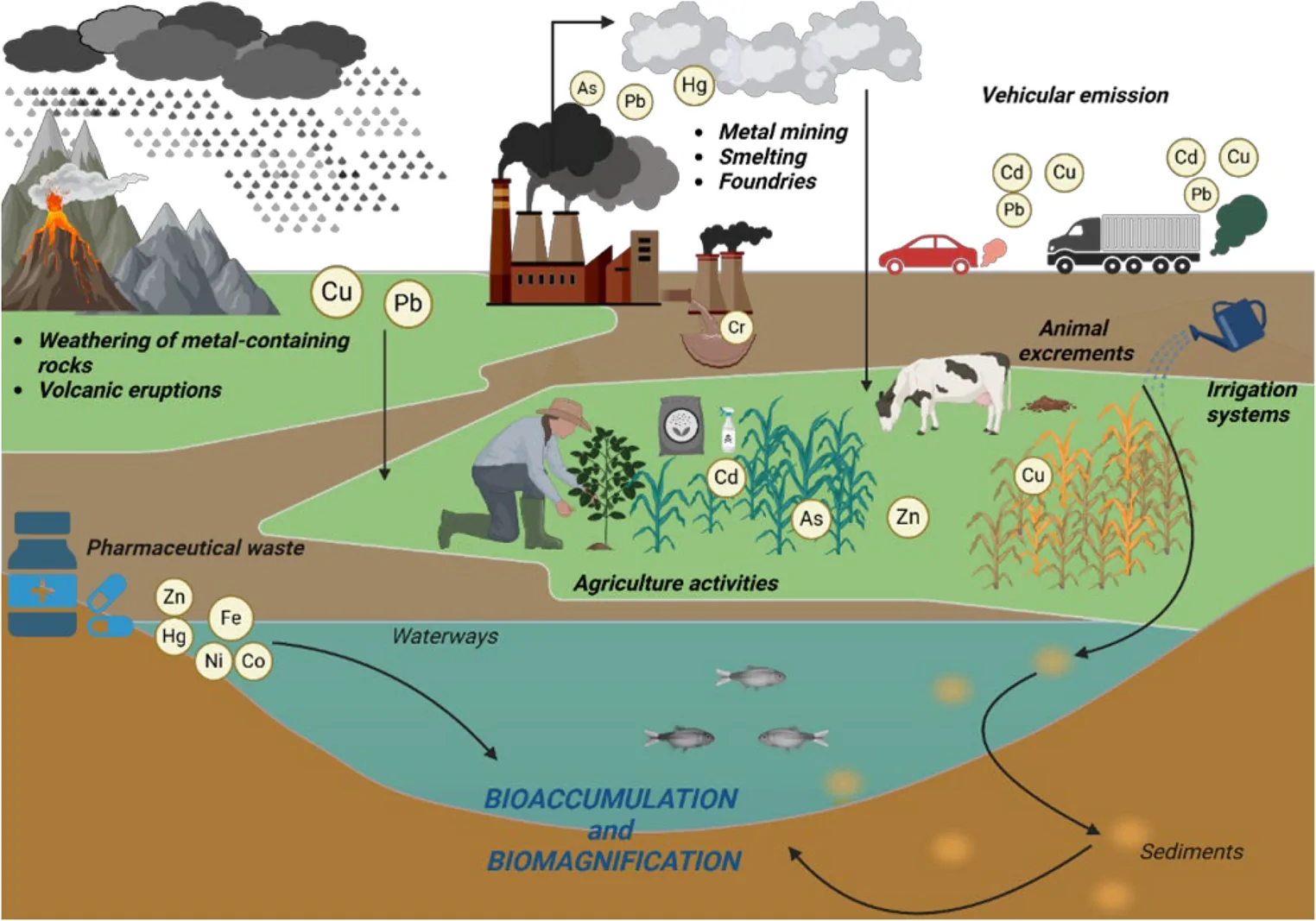
Natural and anthropogenic sources of potentially toxic elements (PTEs) and their distribution pathways in the environment

Potentially toxic elements (PTEs) represent a persistent category of environmental contaminants found in soil and aquatic ecosystems (Pourret and Hursthouse 2019; Pourret et al. 2021).Since the term PTEs can be too broad without proper clarification, it is used here in an operational sense to refer to chemical elements of environmental concern, mainly metals and metalloids, whose impact depends on their persistence, concentration, chemical speciation, bioavailability, mobility, potential toxicity, and behaviour in environmental matrices (Zhang et al. 2022b). While PTEs naturally occur in the Earth’s crust, their harmful effects on the environment are mainly linked to their accumulation downstream of assiduous human activities such as mining, smelting, industrial emissions, atmospheric deposition, wastewater irrigation, manure, fertilizers, and pesticide application (Wan et al. 2024; Cai et al. 2024; Sachdeva et al. 2025) (Fig. 1).

Because soil is not a closed system but a dynamic and permeable matrix, PTEs can migrate through leaching, erosion and runoff into groundwater, rivers, and marine ecosystems (Mor et al. 2022; Jonjev et al. 2024). Once introduced into aquatic environments, these pollutants may be taken up by aquatic primary producers and invertebrates, entering food webs and undergoing bioaccumulation, for some elements and chemical forms, trophic transfer may also lead to biomagnification (Córdoba-Tovar et al. 2022; Danovaro et al. 2023). This close soil-water connection makes PTE contamination a cross-compartment issue and underscores the need for remediation strategies that consider not only contaminant removal, but also mobility, speciation, bioavailability, and ecological risk of metals. Microbial bioremediation has been widely used for managing PTE-contaminated matrices, as bacteria and fungi, either as isolated strains or as members of complex microbial communities, can affect the fate of metals and metalloids through biosorption, intracellular accumulation, redox transformation, biomineralization, precipitation, chelation, and sequestration mediated by biofilms or extracellular polymeric substances (Pande et al. 2022).

Experimental studies support these mechanisms, including Cr(VI) removal by indigenous chromium-resistant bacteria, Cr(VI) reduction mediated by microbial exudates, and competitive Pb(II)/Cd(II) bioaccumulation by *Aspergillus niger* (Dogan et al. 2011; Qiu et al. 2021; Nacer et al. 2021).

However, the relevance and efficiency of these mechanisms remain highly context-dependent, being influenced by matrix properties, pH, redox conditions, metal speciation, bioavailability, organic matter, competing ions, contaminant concentration, and microbial community structure (Ayala-Muñoz et al. 2020; Pande et al. 2022; Zhou et al. 2023; Ding et al. 2024).

The translation of laboratory evidence to field-relevant applications remains a major obstacle in microbial bioremediation of PTE-contaminated matrices. Although tolerance, biosorption, transformation, or removal can be demonstrated under controlled conditions, these findings are not always directly transferable to natural soils, sediments, or waters, where microbial interactions, nutrient availability, spatial heterogeneity, fluctuating environmental and geochemical conditions and microbial community structure can substantially influence process performance (Dey et al. 2022; Silva et al. 2024). Accordingly, biological and physicochemical remediation technologies should be compared not only by initial efficiency, but also by the persistence of the effect, applicability at pilot or field scale, and the risk that treatment-induced changes may promote contaminant remobilization and secondary contamination. The distinction between removal and immobilization is particularly relevant. Immobilization confines contaminants within the treated matrix rather than eliminating them and may therefore be reversible if the conditions maintaining the less mobile fractions change. At the pilot scale, Pasciucco et al. (2024) examined treatment duration and energy consumption in an electrokinetic system applied to dredged marine sediments. The authors reported that higher pH near the cathode promoted metal precipitation and accumulation, whereas longer treatment and effective pH reduction improved metal removal. Extending the experiment from 70 to 95 daysincreased energy consumption from 1547 to 2705 kWh m-³. The increased energy demand associated with the longer treatment highlights an operational constraint that must be considered when transferring this physicochemical technology to larger-scale applications (Pasciucco et al. 2024). Field applications of biological treatments present a different set of constraints, related to the persistence of microbial activity and its response to environmental variability. Among bacterial approaches, Jiang et al. (2026) investigated the in situ bioaugmentation of U(VI)-contaminated groundwater with the bacterium *Desulfovibrio vulgaris* UR1. The field pilot experiment achieved more than 90% U(VI) removal within three days and maintained this performance for more than 60 days. Separate controlled experiments linked enhanced U(VI) reduction to biofilm formation and extracellular polymeric substance production. These mechanistic findings were considered separately from the field-performance data. The maintenance of U(VI) removal over the 60-day monitoring period supports the feasibility of bacterial bioaugmentation under field conditions, although longer-term persistence was not assessed (Jiang et al. 2026). Whether a field-scale effect can be maintained when environmental conditions change was addressed more directly by Liu et al. (2024), who combined iron-oxidizing bacteria with organic fertilizer in a contaminated calcareous soil. The combined treatment transformed available As and Cd into more stable fractions, including residual forms and associations with Fe-Mn/Al oxides. Stabilization efficiencies determined through the Toxicity Characteristic Leaching Procedure (TCLP) reached their maximum after 120 days. The authors nevertheless reported secondary release during the stabilization process and found that pH fluctuations disrupted the formation of iron-calcium complexes. The stabilization of As and Cd was therefore not constant over time, indicating that changes in soil conditions may favour renewed PTE mobilization and generate a risk of secondary contamination. Accordingly, the authors identified long-term monitoring and model development as necessary to characterize the stability of the remediation process (Liu et al. 2024). Biological stabilization under field conditions is not limited to bacteria. González-Chávez et al. (2019) investigated the contribution of arbuscular mycorrhizal fungi to a mycorrhizal-assisted phytostabilization approach. Four arbuscular mycorrhizal fungal inocula were separately applied to *Ricinus communis* plants established on amended soil at a former lead-acid battery-recycling site and monitored for 15 months. The inocula produced species-specific effects: *Acaulospora* sp. modified rhizosphere pH and decreased foliar Pb concentrations 3.5-fold, whereas *Funneliformis mosseae* BEG25 decreased soil Pb availability threefold relative to non-inoculated plants (González-Chávez et al. 2019). The authors presented this approach as mycorrhizal-assisted phytostabilization rather than Pb removal from the soil (González-Chávez et al. 2019). The species-specific responses observed during the 15-month field experiment support the contribution of selected mycorrhizal fungi to reducing Pb availability or its transfer to plant shoots. However, because Pb was stabilized rather than removed from the contaminated soil, the persistence of this effect beyond the monitoring period remains to be established (González-Chávez et al. 2019). Although these studies address different PTEs and environmental matrices and do not permit a direct quantitative comparison, they show that initial remediation efficiency alone is insufficient to evaluate either biological or physicochemical technologies. The electrokinetic study illustrates how scale-up may be affected by treatment duration and energy demand, whereas the bacterial and fungal studies show that field performance must be interpreted in relation to the duration of the observed effect and, when immobilization is the remediation endpoint, to the environmental stability of the resulting PTE fractions. Scalability, persistence and the risk of secondary contamination are therefore interdependent aspects of remediation performance, particularly when PTEs are immobilized rather than removed (González-Chávez et al. 2019; Pasciucco et al. 2024; Liu et al. 2024; Jiang et al. 2026).

A key challenge is, therefore, to move beyond descriptive strain selection and removal assays toward a clearer understanding of which molecular mechanisms are triggered in microorganisms under PTE stress conditions and which of these mechanisms are functionally relevant for the treatment of contaminated matrices (Lu et al. 2017; Ayala-Muñoz et al. 2020).

These examples illustrate how, in recent years, numerous applications have effectively employed various microorganisms to complement remediation strategies with bioremediation strategies. To optimize treatment efficiency, contain costs, and reduce the impact of potential unexpected side effects, demand in recent years has focused on selecting microbial strains (bacteria, fungi, algae) based on their ability to adapt to PTEs in various environmental matrices, and on understanding the molecular mechanisms underlying such adaptation, which can be used to optimize bioremediation strategies.

Omics technologies can contribute to this mechanistic understanding, although they are not sufficient on their own to predict bioremediation performance under real environmental conditions. By providing complementary information on genetic potential, regulatory activation, protein-level responses, and biochemical outcomes, omics approaches can help link microbial traits with observed processes of tolerance, transformation, immobilization, or removal (Ayala-Muñoz et al. 2020; Salaskar et al. 2022). However, detecting genes, transcripts, proteins, or metabolites does not necessarily demonstrate effective PTE remediation. Omics-based interpretations should therefore be integrated with measurements of metal(loid) concentration, speciation, bioavailability, microbial activity, and process performance, such as removal, immobilization, or transformation efficiency (Helfrich et al. 2024).

This review critically evaluates the distinct and complementary contributions of genomics, transcriptomics, proteomics, and metabolomics to investigations of microbial responses to PTEs (metals and metalloids) in bacteria, fungi, and microbial communities in soil and aquatic systems. Rather than offering a catalogue of omics studies, the review compares the type and level of evidence produced by each approach, encompassing encoded genetic potential, transcriptional regulation, protein-level responses, and biochemical outputs. Special emphasis is placed on methodological and interpretive limitations unique to each omics layer, including reference database and annotation constraints, sample and matrix complexity, and the disparity between detecting molecular responses and demonstrating functional bioremediation outcomes. Additionally, the review assesses how the different omics layers complement one another and how their integration with phenotypic, chemical, and geochemical measurements can help distinguish microbial tolerance and stress adaptation from effective PTE biosorption, bioaccumulation, transformation, immobilization, removal, or recovery. Moreover, it addresses the challenges of translating laboratory-derived molecular evidence into environmentally relevant, practically applicable bioremediation strategies, emphasizing the feasibility of moving from *ex situ* to in situ applications. Finally, the role of each omic layer and the synergy of their combination across the phases of a real bioremediation project (screening, implementation, monitoring), has also be addressed, as a perspective of effective and concrete applications.

## General PTEs’ bioremediation mechanisms

Bacteria and fungi are among widely employed microorganisms in environmental biotechnology for the remediation of PTEs- contaminated matrices because of their adaptability, metabolic diversity and ability to interact with metals and metalloids through multiple physicochemical and biological processes (Firincă et al. 2023; Ding et al. 2024).

Their capacity to tolerate, transform, immobilize or remove PTEs can be operationally classified into two major classes of mechanisms: passive (metabolism-independent) and active (metabolism-dependent) processes; however, these categories are not always mutually exclusive, as several remediation outcomes result from the combined contribution of surface interactions, microbial metabolism and extracellular products. Passive mechanisms operate independently of metabolism and are mainly based on biosorption, i.e., the physicochemical binding of PTEs metal(loid) ions to microbial surfaces or extracellular matrices. These processes encompass ion exchange, adsorption, coordination and complexation and can remain effective even when non-living biomass or isolated extracellular polymers are used (Medina-Armijo et al. 2024; Munir Ahamed et al. 2024).

In bacteria, metal binding is mediated by negatively charged, metal-reactive functional groups, including carboxyl, phosphate, hydroxyl, and amino groups associated with peptidoglycan, teichoic acids, lipopolysaccharides, surface proteins, and extracellular polymeric substances. In fungi, chitin, β-glucans, mannoproteins, melanin and other cell-wall or extracellular components provide numerous potential metal-binding sites. Collectively, these structural traits enable both living and dead biomass to bind PTEs such as Cu, Zn, Pb, Cd, and Cr although sorption efficiency depends on the microorganism, metal species, pH, competing ions and biomass characteristics (Medina-Armijo et al. 2024; Munir Ahamed et al. 2024). Active mechanisms require metabolically active cells and involve intracellular transport, transformation, or detoxification of PTEs (Wang et al. 2021). This broad category includes several distinct processes, such as bioaccumulation, biotransformation, biomineralization, and other strategies (Ding et al. 2024).

**Bioaccumulation** is, in detail, the uptake and storage of metals within cells via specific membrane transport systems. Once internalized, metal ions can be complexed by intracellular ligands or compartmentalized within vacuoles or other cellular structures, thereby reducing their environmental mobility (Shi et al. 2024). However, intracellular accumulation results in effective removal from the contaminated matrix only when the metal-enriched biomass is retained, separated, or subsequently recovered; cell death and lysis may otherwise promote remobilization.

**Biotransformation** consists of enzymatically mediated reactions, including oxidation, reduction, methylation or demethylation, that alter metal speciation and consequently modify mobility, bioavailability, toxicity or volatility (Ding et al. 2024).

**Bioprecipitation**, or **biomineralization**, involves microbial formation of insoluble mineral phases, including carbonates, phosphates, sulfides, and oxides, which can incorporate or co-precipitate metal ions. Ureolytic biomineralization, for example, can promote carbonate formation and immobilize metals such as Cu, Pb and Zn (Hu et al. 2024b). Nevertheless, the long-term stability of the resulting minerals depends on environmental conditions, particularly pH, redox potential and the presence of competing ligands.

Additional strategies include producing extracellular chelators, such as organic acids and siderophores; actively exporting metal ions through efflux systems; and intracellular chelation by glutathione, phytochelatins, or metallothioneins. Organic acids and phosphate-releasing metabolism can promote metal precipitation and stabilization, as observed for Pb and Cd, whereas siderophores and other metallophores may enhance metal mobilization and mineral weathering rather than immobilization (Yang et al. 2023; Blanco Nouche et al. 2023). Intracellular metallothioneins and related ligands can instead reduce metal toxicity through chelation and sequestration, as demonstrated for Cd and Zn in fungi (Sácký et al. 2022).

**Fig. 2:**
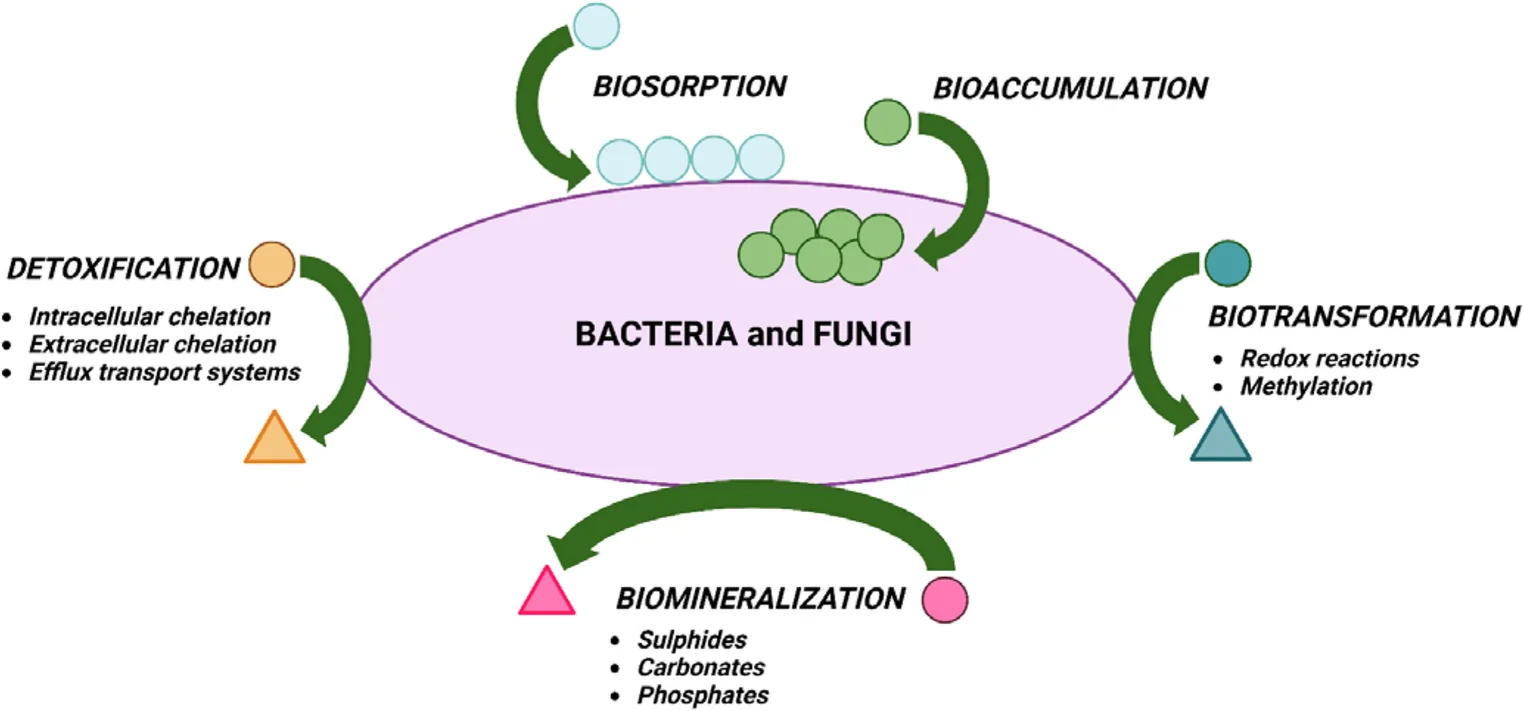
Bioremediation mechanisms of PTEs by bacteria and fungi: passive (biosorption) and active processes (bioaccumulation, biotransformation, biomineralization and detoxification)

The principal microbial bioremediation mechanisms are summarized in Fig. 2.

## Application strategies

*Ex situ* and in situ approaches differ not only in where remediation takes place, but also in the degree of control available over the process. In *ex situ* strategies, contaminated soil or water is transferred to an engineered unit, where operating conditions can be regulated and monitored more directly. Conversely, in situ treatments are conducted directly on the area to be reclaimed. They are therefore largely influenced by contaminant distribution, permeability, amendment transport, and local conditions such as pH, redox potential, and temperature. These constraints must be considered in relation to the PTE involved and the intended remediation endpoint, such as removal, redox transformation, or immobilization.

Omics data cannot determine the choice between *ex situ* and in situ treatment on their own. However, they can show how microbial communities and functions respond to operating conditions and site-specific constraints, informing the setup of both approaches, though in different timeframes and modalities.

The purpose and insights derived from omics analyses typically refer to adaptation studies in laboratory-reproduced environments, where every condition (type and concentration of pollutants, pH, temperature, atmosphere, and growth conditions) is monitored so that molecular analysis, especially at the level of macromolecules most sensitive to environmental impact (RNA, proteins, and metabolites), provides a snapshot of a perfectly controlled environment. Given the variability of transcriptomes, proteomes, and consequently metabolomes, a fully controlled process chain is the only condition that guarantees correct data interpretation. The results thus obtained concerning the molecular response induced/controlled by specific stress conditions, such as those induced by high concentrations of PTEs, can reveal which processes can contribute to specific types of bioremediation. Conversely, genomics differs from other omics because the molecular entity it targets (i.e., the genome) is, in the short term, indifferent to simultaneous physical, chemical, and physicochemical variation in environmental parameters. However, genomics studies are essential for typing microbial communities living in the territory and for implementing specific in situ strategies. These findings can therefore enable targeted in situ applications by selecting specific microorganisms and their communities, implemented through the presence of recombinant enzymes/proteins (as suggested by proteomics) or the large-scale synthesis/purification of bioactive molecules (as suggested by metabolomics). In every case, molecular results must be interpreted in light of other parameters such as PTE concentrations, speciation, mineralogical changes, and process performance.

Van Landuyt et al. (2021) monitored an operational denitrifying bioreactor treating metallurgical wastewater containing arsenic and nitrate, for one year using culture-independent sequencing and multivariate analysis. Denitrification remained relatively stable, although time, sampling depth, pH, and influent composition affected microbial community structure (Van Landuyt et al. 2021). The community was then transferred to small-scale simulation reactors to examine its response to increasing As(V) and As(III) concentrations before introducing changes into the operating system (Van Landuyt et al. 2021). This approach showed how microbial community analysis can support the evaluation of process stability under controlled *ex situ* conditions.

Despite the difficulties related to the impossibility of controlling all the parameters that can influence the results of omics investigations, such as transcriptomics, proteomics, and metabolomics, to date there are many examples of their applications in the analysis and standardization of in situ bioremediation strategies, integrated with the monitoring of many other chemical and physical parameters and with organic and inorganic matrix composition.

Wilkins et al. (2009) applied a proteogenomic approach during acetate-stimulated in situ remediation of a uranium-contaminated aquifer in Rifle, Colorado. The analysis examined the strain composition and physiology of the dominant “*Geobacter*” populations involved in the reduction of soluble U(VI) to insoluble U(IV); in addition, proteomic data, integrated by measurements of uranium concentrations in groundwater, provided information on the processes active in microbial populations associated with uranium transformation (Wilkins et al. 2009).

Lian et al. (2024) also addressed groundwater contamination by uranium by evaluating biostimulation and bioaugmentation approaches. High-throughput sequencing showed increased relative abundance of *Desulfosporosinus* and *Sulfurospirillum* after treatment, and parameters such as initial uranium concentration, the presence of a carbonate-rich matrix, and temperatures below 15 °C were identified as major factors affecting performance and treatment duration. However, the reported results are of considerable importance, since bioaugmentation achieved 91.26% uranium removal after 120 days and reduced the treatment period by 30–60 days relative to biostimulation, while reaching a similar final efficiency (Lian et al. 2024).

Pérez-de-Mora et al. (2024) stimulated native sulfate-reducing bacteria at an industrial site contaminated with As, Ni, and Zn by injecting emulsified vegetable oil and potassium bicarbonate. Metal and metalloid concentrations declined markedly during the first year and remained low throughout the 34-month monitoring period. Amplicon sequencing and shotgun metagenomics identified *Desulfosporosinus* as the major sulfate-reducing genus, while mineralogical and sulfate-isotopic analyses confirmed the formation of biogenic metal sulfides. Integrating these measurements linked the microbial response to metal precipitation and reduced contaminant mobility in groundwater (Pérez-de-Mora et al. 2024).

Together, these studies show how Omics data cannot determine the choice between *ex situ* and in situ treatment on their own. However, they can provide useful information on how microbial communities respond to operating conditions and site-specific constraints. In *ex situ* systems, they can track changes in the microbial community during treatment by accurately monitoring each environmental parameter and the biological response. In situ applications are more complex to control and to determine optimal applicability downstream of a preliminary ex situ experimental setup.

However, all omics data must be considered alongside PTE concentrations and speciation, mineralogical changes, and treatment performance. Community profiling, proteogenomics, and sequencing approaches identified microorganisms associated with arsenic tolerance, uranium reduction, and metal sulfide precipitation. However, interpreting in situ performance still requires integration with chemical, geochemical, and mineralogical measurements, particularly in relation to temperature, carbonate concentration, pH, redox conditions, sulfate availability, and aquifer properties.

## Potential of omics approaches in outlining strategies in ptes’ bioremediation

A comprehensive understanding of the mechanisms underlying microbial responses to PTE exposure is essential for targeted, efficient application of sustainable bioremediation strategies. Unlike organic contaminants, metals and metalloids are persistent and non-degradable; thus, their remediation mainly relies on reducing their mobility, bioavailability, and toxicity, or on promoting their sequestration, immobilization, transformation, or removal from contaminated matrices (Jhariya and Pal 2022). In this context, omics approaches provide valuable tools to investigate the adaptive and detoxification strategies triggered by microorganisms upon this PTE exposure across multiple molecular levels, supporting the selection of promising microorganisms on the basis not only of their genetic potential, but also of the expression of proteins with specific enzymatic or transporter functions relevant to bioremediation practice (Chen et al. 2023; Goff et al. 2023). Each omics layer provides a distinct level of biological information, progressing from encoded functional potential to condition-specific regulatory, protein-level, and biochemical evidence. Genomic analysis represents a first step in omics-based investigations, as it provides a broad overview of the functional repertoire encoded by a microorganism or microbial community and defines its potential capacities and applicability (Li et al. 2023). In particular, in bacteria, the recurrence of recognizable metal-resistance determinants and gene clusters across taxa can reveal genomic patterns suggestive of potential functions, including metal sensing, transport and efflux, as well as element-specific detoxification or redox transformation processes (Mahbub et al. 2023; Adhikary et al. 2024). These patterns can support preliminary selection of candidate microorganisms and the formulation of mechanistic hypotheses to investigate through downstream analyses. However, the presence of specific metal-resistance genes or other resistance-related determinants in the genome does not necessarily indicate that they are expressed, translated into functional proteins, or directly responsible for the observed phenotype under a given environmental condition (Ayala-Muñoz et al. 2020). Therefore, genomics reaches its predictive limit when gene or pathway presence is used to infer condition-specific activation, protein activity, causal involvement in the phenotype, or measurable PTE transformation, immobilization, or removal (Ayala-Muñoz et al. 2020; Sharma et al. 2022). Transcriptomics provides the next regulatory layer by revealing whether candidate genes and pathways are activated or repressed under defined PTE-exposure conditions; proteomics and metabolomics then validate these findings by identifying the proteins and metabolites produced, accumulated, or released under stress conditions. The strength of omics-based interpretation therefore comes from integrating these molecular layers, rather than replacing one approach with another.

For example, in biosorption studies, secretomic and proteomic analyses may reveal extracellular or surface-associated proteins potentially involved in metal binding or sequestration. At the same time, metabolite profiling may identify low-molecular-weight ligands associated with these interactions. These molecular data can be integrated with sorption assays, quantification of the PTE associated with the biomass, and physicochemical characterization of the biomass–metal interaction to support the interpretation of the process (Zhang et al. 2023; Medina-Armijo et al. 2024; Munir Ahamed et al. 2024) and to suggest specific (bio) molecule classes with properties useful in the immobilization, transformation, or precipitation of PTEs.

Zeiner et al. (2021), for instance, carried out fungal secretomic analysis combined with activity assays and identified extracellular enzymes with protein-dependent Mn(II) oxidation properties (Zeiner et al. 2021).

Similarly, in immobilization studies, molecular data from omics-integrated approaches have to be integrated with measurements of PTE mobility or bioavailability and with chemical, microscopic, or mineralogical characterization of the resulting solid phases, to achieve a comprehensive readout. Wang et al. (2019) integrated proteomics with physicochemical analyses in *Pleurotus ostreatus* to explain a combined Pb-removal mechanism involving biosorption, bioaccumulation, and precipitation.

**Fig. 3:**
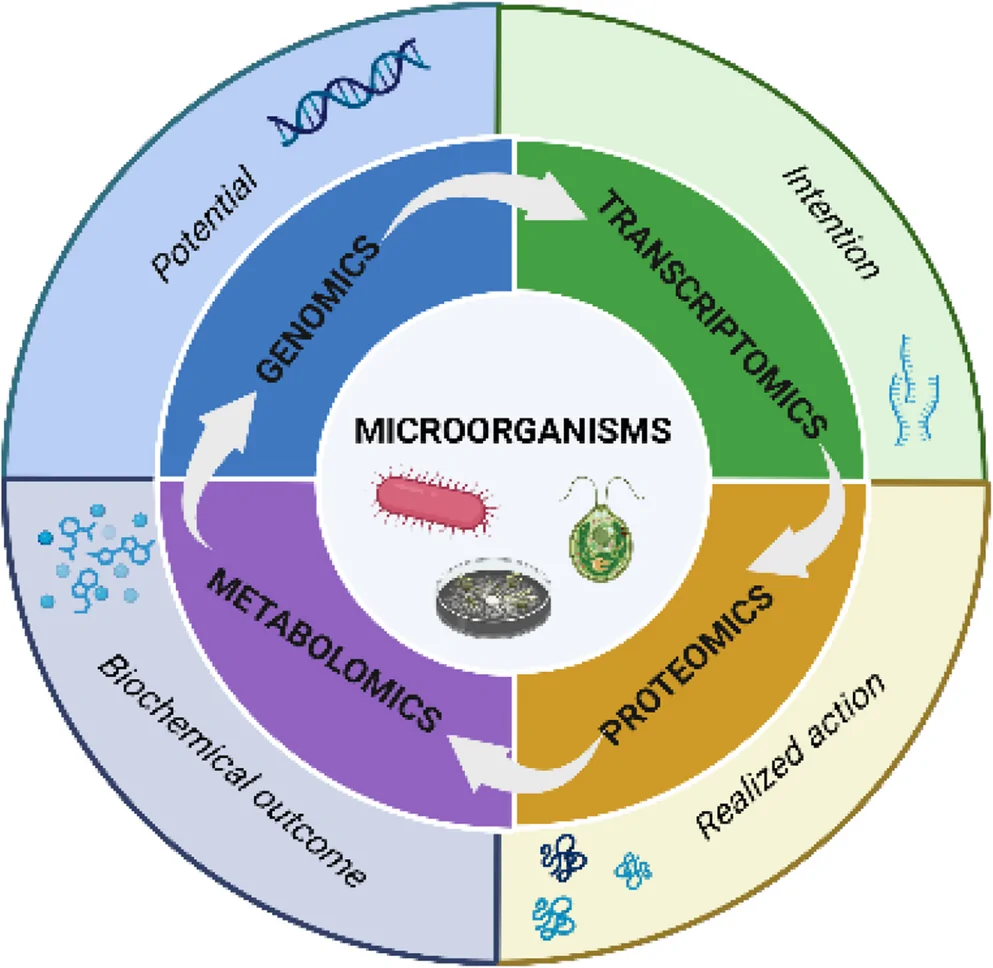
Schematic overview of the four main omics layers used to interpret microbial responses to PTE exposure

Figure 3 graphically represents the interconnections and applications of these approaches. At the same time, Table 1 provides a comparative overview of the information provided, main limitations, scalability, and field readiness of the four omics approaches.

**Table 1:** Comparative overview of omics approaches in microbial PTE bioremediation

Omics layer	What is measured	Mechanistic information	Main limitations	Relative cost and scalability^a^	Field-readiness^b^
Genomics (Bharti and Grimm 2021; Li et al. 2023; Vitale et al. 2025)	Genomes, metagenomes and genes associated with metal resistance or transformation	Encoded potential for PTE tolerance, adaptation, transport, redox transformation and biomineralization	Gene detection does not establish expression or activity; assembly completeness and genome annotation quality affect the current and also other omics applications.	Sequencing depth and assembly complexity for single isolates and communities require massive analytical, computational, and economic effort.	Useful for isolate screening and baseline community profiling; functional validation remains necessary.
Transcriptomics (Mukherjee and Reddy 2020; Lara et al. 2021; Chen et al. 2023)	Transcript sequencing and abundance variation under PTE-exposure conditions	Genes and pathways activated or repressed during PTE exposure	RNA instability due to a short half-life; community complexity and the low correspondence between transcript and protein complicate interpretation	Rapid degradation affects RNA quality; sequencing depth and assembly complexity of single and of communities require massive analytical, computational, and economic efforts	Field application requires immediate sample preservation and a consistent sampling design
Proteomics (Lin et al. 2021; Blakeley-Ruiz and Kleiner 2022; Nebauer et al. 2024)	Identification and quantification of proteins differentially abundant in microbial communities grown in the presence of PTEs	Protein-level responses associated with metal resistance, redox balance and maintenance of microbial functions; pathways activated or repressed during PTE exposure	Protein recovery and identification depend on sample processing, LC-MS/MS analysis, and the availability of complete, annotated sequence-protein/genome databases.	Sample preparation, expense of mass-spectrometric workflow; LC–MS/MS acquisition and specialized data analysis constrain throughput;	Applicable to complex microbial communities, although results remain sensitive to sample processing and database construction
Metabolomics (Goff et al. 2023; Thukral et al. 2023; Tian et al. 2024)	Low-molecular-weight metabolites and metabolite profiles	Biochemical changes associated with metal stress, redox balance and metabolic adaptation	Metabolite annotation and source attribution remain difficult, particularly in complex environmental samples.	Untargeted workflows require broad MS acquisition and extensive data processing; targeted assays examine predefined metabolites.	Untargeted profiling is mainly discovery-oriented; targeted workflows allow quantitative analysis of selected metabolites.

ᵃRelative cost and scalability are expressed qualitatively because they depend on sample type and complexity, analytical depth, replication, platform availability, and bioinformatic requirements

ᵇField-readiness refers to the practical suitability of each omics approach for environmental sampling or process monitoring and does not imply that omics data alone demonstrate effective remediation

### Genomics as the starting point for exploring microbial PTE bioremediation potential

Genomic approaches provide an initial assessment of the taxonomic identity and functional potential of microorganisms and microbial communities. Marker-gene sequencing is commonly used for taxonomic identification and community profiling. 16 S rRNA gene analysis primarily targets bacteria and archaea, whereas ITS, 18 S rRNA, LSU/28S, and other barcode regions are used for fungi and other eukaryotes (Bharti and Grimm 2021; Forsman et al. 2022). Whole-genome sequencing (WGS), in contrast, provides access to the broader genetic repertoire of individual microorganisms, including Metal Resistance Genes (MRGs), resistance clusters, Mobile Genetic Elements (MGEs), transport systems, and redox-related determinants. Comparative genomics can distinguish shared and strain-specific traits, whereas metagenomics extends this analysis to the resistome, the taxonomic distribution of MRGs, and their association with MGEs in microbial communities (Mahbub et al. 2023). The conserved and diverse nature of these systems has led to the development of dedicated resources, such as the Metal Resistance collection developed by Martiny et al. (2023) for MRG identification. Genomic evidence is mainly predictive because gene presence does not necessarily correlate with expression or functional involvement under environmental conditions. Therefore, genomic analysis serves as a preliminary screening tool to identify microorganisms with genetic traits indicating possible PTE tolerance or bioremediation potential, helping select candidates for further omics and phenotypic analyses (Vitale et al. 2025).

From a technical and analytical perspective, genomic investigations of PTE-adapted microorganisms are mainly based on whole-genome sequencing (WGS) for isolates and shotgun metagenomic sequencing for complex microbial communities. Typical workflows include (i) controlled sampling or isolation of microorganisms from PTE-exposed environments, followed by high-quality DNA extraction and quality assessment; (ii) library preparation and high-throughput sequencing, using short-read platforms and increasingly long-read technologies, such as PacBio HiFi and Oxford Nanopore, either independently or in hybrid strategies; (iii) read quality control, genome or metagenome assembly, polishing and, in metagenomic studies, genome binning to reconstruct metagenome-assembled genomes (MAGs); (iv) structural and functional genome annotation, including the identification of metal-resistance genes (MRGs), transporters, redox-related determinants, stress-response genes and mobile genetic elements; and (v) comparative genomic, phylogenomic and resistome analyses to identify conserved and strain-specific determinants, reconstruct their genomic context and investigate their taxonomic distribution and potential mobility. Long-read and hybrid approaches can improve genome contiguity and help resolve repetitive regions, plasmids, and other mobile genetic elements, although their relative performance depends on biological complexity and the sequencing strategy adopted (Alberdi et al. 2022; Kim et al. 2024; Eisenhofer et al. 2024).

In bacteria, MRGs are often organized into recognizable functionally coordinated modules or operon-like clusters, including systems such as *cop*, *cus*, *czc*, *ars*, *mer* and *chr* (Lan et al. 2023; Adhikary et al. 2024). In detail, *cop* and *cus* systems are primarily linked to copper sensing, homeostasis, and export; *czc* systems participate in Co–Zn–Cd resistance; *ars* modules are involved in arsenic transformation and efflux; *mer* systems are associated with mercury detoxification; and *chr* determinants are involved in chromate resistance, transport, or reduction (Lan et al. 2023; Adhikary et al. 2024).

This grouping does not represent a comprehensive formal classification, and the categories are not mutually exclusive, as multiple functions may coexist within the same resistance module or genome (Oleńska et al. 2025).

Metal transport, homeostasis, and efflux are recurring components of bacterial resistance systems. P-type ATPases use ATP hydrolysis to transport metal ions across the cytoplasmic membrane, thereby contributing to the regulation of intracellular metal concentrations, homeostasis, and detoxification (Adhikary et al. 2024; Oleńska et al. 2025). HME-RND systems form multicomponent efflux complexes that mediate metal export across the envelope of Gram-negative bacteria. This family includes well-characterized metal-resistance systems such as Czc-type transporters, whereas Cus-type systems are related RND-based complexes that primarily mediate copper and silver homeostasis and export (Bodilis et al. 2024; Adhikary et al. 2024). Element-specific transformation and detoxification constitute another recurring function. These systems may encode reductases, oxidases, or other enzymes that modify PTE speciation, toxicity, or mobility, as illustrated by ars-, mer-, and chr-associated determinants (Lan et al. 2023; Adhikary et al. 2024). Additionally, accessory protective systems often support transport and detoxification processes. Thiol- and NADPH-dependent pathways, along with defenses against reactive oxygen species and DNA repair mechanisms, may not directly target metals, but instead protect cells from oxidative, redox, and genotoxic damage caused by metal exposure or transformation (Ouertani et al. 2020; Adhikary et al. 2024; Huang et al. 2024). These bacterial resistance systems may also be associated with plasmids, transposons, integrons, integrative and conjugative elements, genomic islands, and other mobile genetic elements. Their presence within mobile genomic regions likely aids their acquisition and spread through horizontal gene transfer (Lan et al. 2023; Mahbub et al. 2023). Along with genetic mobility, selective pressure in contaminated environments might drive the repeated emergence of similar resistance modules across different taxa and ecological settings (Lan et al. 2023; Mahbub et al. 2023). The recurrent occurrence of genomic transport, homeostasis, and efflux determinants directed toward different PTEs within the same genome supports adaptation to environments containing mixtures of metals. Genome annotation of the plant growth-promoting bacterium *Gluconacetobacter azotocaptans* DS1 allowed the identification of several groups of determinants potentially involved in metal handling. Copper transport and homeostasis were associated with copA, ctpA and an ATP7-like determinant, whereas mntD was associated with manganese homeostasis. The HME-RND components czcA and czcC were linked to Co–Zn–Cd efflux, while *znuB* and *SOD1* were associated with zinc metabolism (Mukhtar et al. 2021).

Another recurrent pattern involves element-specific redox transformations combined with transport, antioxidant, and DNA repair functions that support cellular survival during PTE exposure. Studies of chromium-resistant bacteria illustrate how strains exposed to the same element may converge on Cr(VI) transformation while relying on different combinations of supporting determinants. In *Pediococcus acidilactici* 13 − 7, which displayed Cr(VI)-reducing capacity, genomic analysis identified *chrR* together with the redox-related determinants *nqo1*,* frdA* and *gshR *(Huang et al. 2024). *ChrR* was associated with chromate reduction, whereas NAD(P)H-dependent electron-transfer functions and glutathione reductase may support the reducing environment and redox balance required during Cr(VI) transformation (Huang et al. 2024).

A broader multistep configuration was identified in *Microbacterium metallidurans* TL13. Sulfate-permease systems were proposed to facilitate chromate entry because of the structural similarity between chromate and sulfate, whereas *chrA* encodes a chromate-efflux transporter that limits intracellular accumulation. Thioredoxin reductase- and NADPH-dependent functions have been proposed to contribute to Cr(VI) reduction, while superoxide dismutase, catalase, peroxidase, and glutathione-related systems may protect against oxidative stress. DNA-repair determinants belonging to the *RecA*,* RecG*, and *RecBCD* systems were also identified (Ouertani et al. 2020). Thus, *P. acidilactici* 13 − 7 and *M. metallidurans* TL13 converge on Cr(VI) reduction and cellular protection but differ in the organization and relative contribution of uptake, efflux, redox, antioxidant and DNA-repair determinants. These differences illustrate how a recurrent functional response can be implemented through distinct strain-specific genomic configurations (Ouertani et al. 2020; Huang et al. 2024).

At broader ecological and community scales, metagenomic and comparative genomic analyses can determine how resistance determinants are distributed across taxa and associated with plasmids, genomic islands and other mobile genetic elements (Xie et al. 2023; Mahbub et al. 2023). These approaches therefore provide an ecological perspective on the recurrence, enrichment and potential dissemination of resistance systems under metal-selective pressure (Lan et al. 2023; Goff et al. 2024).

Goff et al. (2024), analyzing 1,615 putative MGEs from differently contaminated subsurface environments, found that MGEs from the most polluted sites were enriched in clusters containing multiple MRGs, some associated with conjugative elements (Goff et al. 2024). Zhao et al. (2025) observed an increase in the abundance of resistance genes for As, Cd, Cu, Zn, and Pb in relation to soil metal contamination levels, suggesting that specific metal pressures can shape the resistome of microbial communities (Zhao et al. 2025). Comparative analysis of *Pantoea eucrina* OB49 and related genomes further identified acquired MGEs associated with metal-resistance determinants, including arsenic-resistance genes (Lekired et al. 2023). Together, these studies indicate that the recurrence of resistance functions across contaminated environments may reflect both selection of pre-existing determinants and their potential mobilization among microbial populations.

Not all genomic traits relevant to bioremediation belong to the classical systems described above. Some microorganisms possess metabolic pathways that directly contribute to PTE immobilization via mineral precipitation. Microbial-induced calcium carbonate precipitation (MICP) differs from cellular efflux or intracellular detoxification because microbial metabolism directly promotes mineral precipitation and metal immobilization (Vitale et al. 2025). In ureolytic MICP, urease-mediated urea hydrolysis increases pH and carbonate availability, favoring calcium carbonate precipitation and PTE immobilization through co-precipitation, incorporation into carbonate minerals, or formation of metal-containing phases (Vitale et al. 2025). In this context, the marine strain *Lysinibacillus sphaericus* PG22 showed genomic and functional potential for metal biomineralization through urease activity (Vitale et al. 2025). Similarly, *Priestia aryabhattai* PMASS1 promoted the precipitation of Pb, Cu and Cd into mineral phases including cerussite, malachite and otavite. Its genome also contained the stress-response determinants *sodA*, *sodC*, *katE*, *btuE* and *trxB*, which were proposed to support survival during metal exposure and biomineralization (Atakpa et al. 2025). In these cases, ureolytic metabolism contributes directly to immobilization, whereas resistance and oxidative-stress determinants primarily support cellular persistence (Atakpa et al. 2025; Vitale et al. 2025).

In fungi, determinants of metal tolerance and homeostasis are also central to the genomic interpretation of PTE responses, but their conservation and organization differ substantially from those of bacterial MRGs. Fungal metal-tolerance determinants are less commonly organized into compact operon-like modules and are more often distributed across diverse eukaryotic gene families and regulatory systems (Gajewska et al. 2022). These systems include CDF transporters, P-type ATPases, ABC transporters, metallothioneins, glutathione- and phytochelatin-related pathways, vacuolar sequestration systems, oxidative-stress responses, and cell-wall or extracellular components involved in metal binding or immobilization (Gajewska et al. 2022).

Horizontal gene transfer also occurs in fungi, but its contribution varies substantially among lineages and ecological lifestyles and is generally less central to interpreting fungal metal tolerance than in bacterial resistance systems (Liu et al. 2025). Fungal metal tolerance is therefore often better described as a distributed, multilayered response involving intracellular sequestration, chelation, compartmentalization, stress adaptation, and cell-surface interactions (Gajewska et al. 2022). Nevertheless, the relative contribution and genomic organization of these functions can vary markedly among species, ranging from broadly distributed polygenic adaptations to compact resistance loci or copy-number variations (Bobrowicz et al. 1997; Zhao et al. 2014; Daghino et al. 2025).

A distributed genomic architecture is illustrated by the ericoid mycorrhizal fungus *Oidiodendron maius*. Comparative resequencing of eight metal-tolerant and ten metal-sensitive isolates revealed extensive divergence across coding and non-coding regions, with approximately one-third of predicted gene models exhibiting high polymorphism. Affected functions include DNA and RNA metabolism, chromatin organization, protein metabolism, energy conversion, mitochondrial processes, and cellular defense (Daghino et al. 2025). Earlier analyses also identified polymorphism at the SOD1 locus and supported a protective role for Cu, Zn-superoxide dismutase during Zn and Cu exposure (Vallino et al. 2009, 2011). Metal tolerance in *O. maius* therefore appears to involve multiple genomic differences and stress-protection functions rather than a single compact resistance locus. More localized fungal architectures have also been identified. In *Saccharomyces cerevisiae*, the contiguous genes *ACR1*, *ACR2*, and ACR3 form a compact arsenic-resistance locus (Bobrowicz et al. 1997). *ACR1* contributes to regulation, *ACR2* encodes an arsenate reductase converting As(V) to As(III), and *ACR3* encodes a membrane protein involved in arsenite export (Wysocki et al. 1997; Mukhopadhyay and Rosen 1998; Ghosh et al. 1999). This locus integrates regulation, element-specific transformation and efflux within a restricted genomic region and therefore represents a fungal example of a compact resistance configuration. Copper homeostasis clearly shows how different fungal species may rely on distinct genomic and regulatory architectures to manage the same metal. In *Cryptococcus neoformans*, copper homeostasis involves the high-affinity copper transporters *CTR1* and *CTR4* and the metallothioneins *CMT1* and *CMT2*, whose expression is coordinated by the transcription factor Cuf1(Garcia-Santamarina et al. 2018). This distributed regulatory system integrates copper acquisition and detoxification across multiple chromosomal loci.

*S. cerevisiae* exhibits a different copper adaptation mechanism. In this species, the *CUP1* gene encodes a copper-binding metallothionein and occurs in tandem repeats, with copy numbers varying among natural isolates (Zhao et al. 2014). An increased number of *CUP1* copies can enhance copper tolerance, but its phenotypic impact depends on the genetic background and other resistance-related loci (Onetto et al. 2023). Therefore, copper tolerance can be achieved either through a broad regulatory network that manages uptake and sequestration, as in *C. neoformans*, or by amplifying a specific metal-binding locus, as in *S. cerevisiae*. These instances demonstrate that fungal responses to the same PTE are highly species-specific and do not follow a uniform genomic pattern. Table 2 summarizes all outcomes from genomic investigations of PTE-induced adaptive mechanisms in bacteria and fungi.

**Table 2:** Major functional classes of genomic determinants associated with potentially toxic element (PTE) adaptation and bioremediation activities in bacteria and fungi

Functional class	Main function	Representative examples	Organism group	Key references
Metal uptake, transport and homeostasis	Metal acquisition, intracellular trafficking and regulation of metal concentrations	*znuB*,* copA*,* ctpA*,* CTR1*,* CTR4*; sulfate-permease systems	Bacteria and fungi	(Garcia-Santamarina et al. 2018; Adhikary et al. 2024; Oleńska et al. 2025)
Metal efflux and detoxification	Reduction of intracellular metal concentrations through active export and detoxification	*czc*,* cus*,* cop*,* chrA*,* ACR3*; P-type ATPases; HME-RND systems	Bacteria and fungi	(Wysocki et al. 1997; Mukhopadhyay and Rosen 1998; Bodilis et al. 2024; Adhikary et al. 2024)
Element-specific transformation	Enzymatic transformation of PTEs into chemical forms with altered toxicity, mobility or bioavailability	*ars*,* mer*,* chrR*,* ACR2*	Bacteria and fungi	(Ghosh et al. 1999; Lan et al. 2023; Adhikary et al. 2024; Huang et al. 2024)
Redox and oxidative-stress defense	Maintenance of cellular redox balance and protection against ROS generated during PTE exposure or transformation	*sodA*,* sodC/SOD1*,* katE*,* gshR*,* trxB*,* nqo1*; peroxidases	Bacteria and fungi	(Vallino et al. 2011; Ouertani et al. 2020; Huang et al. 2024; Atakpa et al. 2025)
Metal sequestration, chelation and immobilization	Binding and sequestration of metals intracellularly or extracellularly, reducing their bioavailability and toxicity	Metallothioneins, phytochelatin-related pathways, *CUP1*,* CMT1*,* CMT2*; cell-wall/extracellular binding	Mainly fungi, also bacteria	(Zhao et al. 2014; Garcia-Santamarina et al. 2018; Gajewska et al. 2022; Onetto et al. 2023)
Biomineralization and mineral precipitation	Metabolic immobilization of PTEs through precipitation, co-precipitation or incorporation into mineral phases	Urease-mediated MICP; urease genes; carbonate precipitation	Mainly bacteria	(Atakpa et al. 2025; Vitale et al. 2025)
DNA repair and genome protection	Repair and protection of DNA from oxidative and genotoxic damage induced by PTE exposure	*recA*,* recG*,* recBCD*	Mainly bacteria	(Ouertani et al. 2020; Adhikary et al. 2024)
Mobile genetic elements and horizontal gene transfer	Acquisition, mobilization and dissemination of PTE-resistance determinants	Plasmids, transposons, integrons, ICEs, genomic islands, conjugative elements	Mainly bacteria, also fungi	(Lekired et al. 2023; Lan et al. 2023; Mahbub et al. 2023; Liu et al. 2025)

The table summarizes the main functional classes of genomic determinants associated with microbial adaptation to PTE exposure. It also highlights representative genes or systems that contribute to the acquisition and dissemination of PTE-resistance determinants, the microbial groups in which they have been described, and key supporting references

Abbreviations: *PTE* potentially toxic element, *HME-RND* heavy-metal efflux resistance–nodulation–division, *ROS* reactive oxygen species, *MICP* microbially induced calcium carbonate precipitation, *ICEs* integrative and conjugative elements

A common challenge across microbial groups is obtaining complete, well-annotated genomes from environmental isolates and complex communities (Haas et al. 2011; Van Der Walt et al. 2017). In bacterial communities, factors such as uneven taxon abundance, strain-level heterogeneity, and shared mobile genetic regions make metagenomic assembly, binning, and assigning resistance genes to specific populations more difficult (Van Der Walt et al. 2017). In fungi and other eukaryotic microorganisms, genome reconstruction and annotation are more complex due to larger genome sizes, intron-rich gene structures, repetitive regions, gene duplications, paralog divergence, and sometimes polyploidy or aneuploidy (Haas et al. 2011; Naranjo-Ortiz et al. 2022). These issues also impact downstream omics analyses, as incomplete or fragmented genome assemblies can decrease transcript mapping accuracy and complicate peptide-to-protein assignments in proteomics.

### Transcriptomics as a dynamic layer of microbial response to PTE exposure

While genomics defines the genetic potential of microorganisms and microbial communities, transcriptomics provides a dynamic view of genes actively transcribed under specific environmental or experimental conditions (Chen et al. 2023). In PTE bioremediation studies, transcriptomic approaches, mainly based on RNA-seq, compare control and metal-exposed conditions, enabling relative quantification of transcript abundance and identification of differentially expressed genes involved in microbial adaptation to metal stress (Lara et al. 2021). Typical workflows include (i) experimental exposure of microorganisms to defined PTE species, concentrations, exposure times and environmental conditions, generally including appropriate untreated controls and biological replicates; (ii) rapid RNA stabilization and extraction, followed by RNA quality assessment and removal or depletion of highly abundant rRNA, with DNase treatment when required; (iii) strand-specific library preparation and reverse transcription of the informative RNA fraction into cDNA, followed by high-throughput sequencing; (iv) read quality control and adapter removal, followed by reference-based mapping or pseudo-alignment, or de novo transcript assembly when suitable reference genomes or transcriptomes are unavailable; and (v) transcript quantification, differential-expression analysis and functional or pathway-level enrichment. In metatranscriptomic studies, reads can additionally be assigned taxonomically and functionally to determine which microorganisms and pathways contribute to the observed transcriptional response. Time-course and dose-response designs can further resolve transient versus persistent transcriptional responses, whereas emerging long-read and single-microorganism RNA-seq approaches offer additional resolution of transcript structures and cell-to-cell heterogeneity (Lott et al. 2017; Shakya et al. 2019; Amarasinghe et al. 2020). This level of analysis helps to distinguish silent genes that are merely present in the genome from those that are transcriptionally activated or repressed in response to PTE exposure (Chen et al. 2023).

In this context, transcriptomics can reveal the activation of metal transport and efflux systems, metal-regulatory networks, redox transformation pathways, oxidative stress responses, DNA repair mechanisms, metabolic reprogramming and processes potentially associated with sequestration, immobilization or detoxification (Rawle et al. 2019; Lara et al. 2021). Depending on the experimental design, RNA-seq can be applied to single isolates, microbial consortia or environmental communities, and can be structured as control-versus-treated comparisons, dose-response experiments or time-course analyses (Ayala-Muñoz et al. 2020; Gan et al. 2024). These approaches are particularly useful because microbial responses to PTEs are highly dynamic: early transcriptional changes may reflect stress perception and regulatory activation, whereas later responses may involve metabolic adaptation, repair mechanisms, biofilm formation, extracellular interactions or long-term tolerance strategies (Miano et al. 2023).

However, transcriptomic data should not be interpreted as direct evidence of functional activity: changes in mRNA abundance indicate transcriptional modulation, but they do not necessarily correspond to protein abundance, enzymatic activity, metabolite production, or measurable metal removal and immobilization (Goff et al. 2023; Zhao et al. 2025). Therefore, transcriptomics represents a dynamic intermediate layer between genomic potential and downstream functional responses, and interpreting it requires integrating proteomic, metabolomic, and phenotypic data to clarify whether transcriptionally activated pathways contribute to PTE tolerance or bioremediation performance (Zhao et al. 2025). In bacteria, transcriptomic responses to PTE exposure are often coordinated by metal-responsive transcription factors, which act as molecular sensors linking metal availability or toxicity to the activation of specific adaptive pathways (Huang et al. 2022). Several metalloregulator families have been described to be involved in this process, including MerR-type regulators, such as MerR, CadR, CueR, ZntR and PbrR, which respond to metals such as Hg(II), Cd(II), Cu(I), Zn(II) and Pb(II), and Fur-family regulators, including Fur, Zur, Mur and Nur, which control iron and other metal-homeostasis networks (Ghataora et al. 2023; Hu et al. 2024a). Other relevant regulatory systems include CsoR/RcnR-type regulators involved in copper and nickel/cobalt responses, DtxR-like regulators such as MntR or IdeR, NikR regulators controlling nickel transport systems, and SmtB/ArsR-type repressors, which regulate genes associated with arsenic, zinc, lead and other metal stress responses. By identifying the genes controlled by these regulators and their differential expression under metal exposure, RNA-seq can reveal how bacteria coordinate metal sensing, efflux, redox transformation, oxidative stress protection and broader metabolic adaptation (Halema et al. 2024).

This regulatory organization is reflected in transcriptomic studies of metal-exposed bacteria, where PTE stress generally induces coordinated responses rather than isolated resistance genes. For example, in the soil bacterium *Sinorhizobium meliloti*, copper exposure induces genes associated with a multicopper oxidase operon, including the multicopper oxidase-encoding gene *cueO*, an outer membrane protein, and copper chaperones. The concomitant induction of an adenylate cyclase suggests that cAMP-mediated signaling may also contribute to the coordination of the copper stress response. In the same organism, zinc exposure specifically activates the P-type ATPase *zntA*, highlighting how transcriptomics can distinguish metal-specific responses within the same bacterial background (Lu et al. 2017). Additional upregulated genes, including regulators, ABC transporters and sulfur-related enzymes, further indicate that metal exposure affects not only resistance determinants but also broader regulatory and metabolic networks.

Transcriptomics is also particularly informative for bacteria involved in redox transformation of metals. In chromium-reducing bacteria such as *Cellulomonas fimi* and *Lysinibacillus fusiformis*, Cr(VI) exposure induces genes encoding transport systems, membrane-associated proteins, reductases, electron transport components, and DNA repair systems. In *C. fimi*, the upregulation of nutrient and metal transporters, including *modABC* and *mntH*, together with osmoprotectant transport systems such as *opuABC*, suggests that chromium stress affects both metal handling and cellular homeostasis. Moreover, genes encoding proton pumps, FMN reductases, quinone oxidoreductases and cytochrome c components may contribute to Cr(VI) reduction to the less toxic Cr(III) form. In contrast, DNA repair and oxidative stress response genes support cell survival during metal-induced damage (Sun et al. 2025). Similarly, *L. fusiformis* shows a broad transcriptional response involving ABC transporters, reductase-coding genes such as ssuE, fnr, qor, and cysJ, and DNA repair genes such as recA and recG, indicating that chromium reduction requires coordinated detoxification, redox balancing, and repair mechanisms (Li et al. 2021).

Other bacterial transcriptomic studies show that PTE stress can also reshape antioxidant defenses and primary metabolism. For instance, *Bacillus altitudinis* HM-12 exposed to high manganese levels modulates catalase systems, replacing standard heme catalases such as KatE/KatX with the manganese-specific catalase YjqC, while also inducing efflux-related genes such as *mneS*, *yceF* and *ykoY*, together with metal-responsive regulators such as CsoR. In *Bacillus velezensis*, cadmium exposure induces genes involved in chemotaxis, siderophore biosynthesis and amino acid metabolism, particularly histidine and arginine pathways. These metabolic changes have been linked to ammonia production through urease activity, pH increase and the biomineralization of cadmium into carbonate minerals such as vaterite (Huang et al. 2022).

In fungi, transcriptomic interpretation should be framed in continuity with the genomic limitations discussed above. Compared with many bacterial systems, fungal transcriptomic analyses are often more affected by incomplete genome annotation and by the structural complexity of eukaryotic genomes, including intron-rich genes, alternative splicing, extended regulatory regions, paralogous gene families and multilayered transcriptional regulation. These features, together with the limited availability of well-curated reference genomes for environmental and non-model species, can complicate read mapping, transcript annotation and the functional interpretation of differentially expressed genes (Ejigu and Jung 2020; Wang et al. 2022).

Moreover, as already discussed, fungal responses to PTE exposure are generally not organized around compact operon-based regulatory modules, as commonly observed in bacteria. Rather, they often involve distributed eukaryotic networks associated with metal sequestration, glutathione-dependent detoxification, vacuolar compartmentalization, oxidative stress response, cell-wall and extracellular interactions, and energy metabolism (Wang et al. 2022).

For instance, *Rhodotorula mucilaginosa* exposed to Pb showed increased glutathione (GSH) production and the formation of intracellular vesicles involved in Pb compartmentalization, suggesting that chelation and intracellular sequestration contribute to fungal metal tolerance (Chen et al. 2022; Shi et al. 2024). Similarly, under Cd stress, the halophilic fungus *Engyodontium album* showed an enhanced transcriptional response involving antioxidant defenses and ROS-scavenging systems to counteract metal-induced oxidative damage (Azam et al. 2025). Transcriptomic analyses of metal-tolerant or hyperaccumulating fungi also indicate that PTE exposure can require extensive metabolic reprogramming. In *Curvularia tsudae*, Pb exposure induced energy-generating pathways, including glycolysis, the pentose phosphate pathway (PPP) and the tricarboxylic acid (TCA) cycle. This metabolic activation may provide the energy and reducing power required to sustain ABC transporters and glutathione-related enzymes, such as glutathione reductase (GR) and glutathione peroxidase (GPX), allowing the fungus to cope with high Pb concentrations while limiting cellular damage (Feng et al. 2023). Similarly, in *Hypomyces chrysospermus*, Cd exposure induced a broad transcriptional response involving genes associated with membrane components, oxidoreductase activity, transport activity, amino acid and carbohydrate metabolism, protein folding/sorting/degradation, antioxidant enzymes, detoxification enzymes and transcription factors. Table 3 summarizes outcomes from transcriptomic studies of adaptive process activation in the presence of PTE accumulation.

**Table 3:** Major functional classes of transcriptionally responsive pathways associated with microbial adaptation to potentially toxic elements (PTEs)

Functional class	Main function	Representative genes, systems or pathways	Organism group	Key references
Metal sensing, regulation and homeostasis	Metal detection and transcriptional regulation of adaptive responses	MerR, Fur, CsoR/RcnR, DtxR, NikR, SmtB/ArsR; *zntA*	Mainly bacteria	(Lu et al. 2017; Ghataora et al. 2023; Hu et al. 2024a)
Metal uptake, transport and efflux	Regulation of metal acquisition, intracellular balance and export	*modABC*,* mntH*,* zntA*; ABC transporters; metal efflux systems	Bacteria and fungi	(Lu et al. 2017; Wang et al. 2022; Sun et al. 2025)
Element-specific transformation and redox metabolism	PTE transformation and maintenance of reducing conditions	*ssuE*,* fnr*,* qor*,* cysJ*; FMN reductases; quinone oxidoreductases	Mainly bacteria	(Li et al. 2021; Sun et al. 2025)
Oxidative stress and antioxidant defense	ROS detoxification and redox homeostasis	Catalases, SOD, GR, GPX, glutathione-related systems	Bacteria and fungi	(Huang et al. 2022; Feng et al. 2023; Azam et al. 2025)
Metal sequestration and intracellular detoxification	Chelation, binding and compartmentalization of PTEs	GSH-related pathways; intracellular vesicles; metallothionein-related systems	Mainly fungi	(Chen et al. 2022; Feng et al. 2023; Shi et al. 2024)
DNA repair and cellular protection	Repair of PTE-induced DNA and cellular damage	*recA*,* recG*; DNA repair pathways; protein quality-control systems	Bacteria and fungi	(Li et al. 2021; Wang et al. 2022; Sun et al. 2025)
Metabolic reprogramming	Redistribution of energy, carbon, nitrogen, and sulfur metabolism to sustain PTE adaptation	Glycolysis, PPP, TCA cycle; amino-acid and sulfur metabolism	Bacteria and fungi	(Huang et al. 2022; Wang et al. 2022; Feng et al. 2023)
Biomineralization and PTE immobilization	Metabolic conversion leading to PTE precipitation and immobilization	Urease-associated metabolism; ammonia production; carbonate precipitation	Mainly bacteria	(Huang et al. 2022)

The table summarizes the main functional classes of genes and pathways whose transcriptional modulation has been associated with microbial responses to PTE exposure. Representative genes, systems or metabolic pathways, the microbial groups in which they have been described, and key supporting references are provided. The classification encompasses both bacterial and fungal responses and highlights the broader metabolic and cellular reprogramming that accompanies the activation of specific PTE-resistance mechanisms

Abbreviations: *ABC* ATP-binding cassette, *FMN* flavin mononucleotide; ROS, reactive oxygen species, *SOD* superoxide dismutase, *GR* glutathione reductase, *GPX* glutathione peroxidase, *GSH* glutathione, *PPP* pentose phosphate pathway, *TCA* tricarboxylic acid cycle

Despite its power, transcriptomics faces intrinsic bottlenecks. RNA is inherently unstable and highly sensitive to degradation, particularly in environmental matrices such as soils and sludge, where humic substances, clay particles and other co‑extracted compounds interfere with extraction, purification and quantification (Zhao 2014). Moreover, transcript abundance does not necessarily correlate with protein levels, enzymatic activity or metabolite production, leaving uncertainty about the actual functional relevance of the observed transcriptional changes (Huang et al. 2022).

For these reasons, transcriptomic data can be interpreted primarily as evidence of transcriptional modulation, indicating which genes and pathways are activated, repressed or rewired under PTE stress, rather than as direct proof of metal removal, immobilization or detoxification (Huo et al. 2022; Zhao et al. 2025). Determining whether these transcriptional programs ultimately translate into effective tolerance or bioremediation requires integration with proteomics, metabolomics, and phenotypic assays, which assess protein expression profiles, biochemical transformations and products, and the actual removal or immobilization of PTEs (Pinu et al. 2019).

### Proteomics as a tool for describing the realized action

Proteomics can complement genomics and transcriptomics by providing information on the proteins and enzymatic systems produced or accumulated under PTE- stress. Compared with genomics and transcriptomics, which describe genetic potential and transcriptional modulation, proteomics can help describe protein-level responses associated with detoxification, resistance, transport, redox balance, sequestration, and extracellular metal interactions (Chakdar et al. 2022).

Despite its potential, proteomics remains less widely applied in environmental bioremediation than in human and model-organism studies, where standardized sample-preparation workflows, extensive reference proteomes, and well-annotated genomes support protein identification, pathway reconstruction, and enrichment analyses (Monti et al. 2019; Deutsch et al. 2026). In environmental and bioremediation contexts, key bottlenecks include difficulties in protein extraction from heterogeneous and complex matrices, the limited availability of complete and well-annotated reference genomes and proteomes for many environmental organisms, and the frequent need to rely on de novo sequence assemblies (Kleikamp et al. 2021; Herruzo-Ruiz et al. 2021; Blakeley-Ruiz and Kleiner 2022). These limitations can reduce protein identification rates, complicate functional annotation, and limit pathway-level interpretation (Lee et al. 2022). Nevertheless, differential proteomics can identify proteins responsive to specific PTE exposure, distinguish tolerance-related responses from pathways more directly linked to bioremediation, and highlight candidate biomarkers or engineering targets (Liao et al. 2020; Fernandez et al. 2023). At a broader scale, metaproteomics extends this functional perspective to complex microbial communities, capturing active ecosystem processes and supporting assessment of field-scale remediation interventions (Lin et al. 2021).

Across microorganisms, proteomics in PTE bioremediation is mainly implemented through mass spectrometry-based workflows that typically include: (i) controlled exposure experiments defining metal species, concentration, exposure time and growth conditions, generally supported by growth or tolerance measurements and, when relevant, scale-up approaches (Goff et al. 2023; Xu et al. 2024); (ii) fraction-specific sampling, based on the physical separation of biomass, such as bacterial pellets, fungal mycelia, from cell-free supernatants (Ferrero-Bordera et al. 2024; Russo et al. 2024) ; (iii) protein extraction and preparation using either gel-based strategies, including SDS-PAGE or 1D/2D electrophoresis followed by in-gel digestion, or gel-free shotgun workflows based on in-solution digestion (Fernandez et al. 2023); (iv) LC-MS/MS acquisition with quantitative analysis, which may be performed using label-free approaches (Barra et al. 2023) or isobaric labelling strategies (Gang et al. 2019); and (v) functional interpretation through protein annotation, pathway reconstruction, enrichment analysis or network-based approaches (Fernandez et al. 2023; Zhang et al. 2023) .

The first two points of the workflow are common to comparative transcriptomic analyses, as they involve setting up preliminary conditions that reproduce or re-propose the stress conditions in comparison with a control.

Most studies distinguish between a cell-associated proteome, derived from harvested biomass, and an extracellular proteome recovered from cell-free supernatants, often called the exoproteome or secretome (Wang et al. 2024a; Ferrero-Bordera et al. 2024). These fractions provide complementary information. The cell-associated proteome mainly reflects internal physiology, including stress and tolerance responses, transport and efflux systems, thiol-based detoxification, redox metabolism, protein homeostasis and metabolic reprogramming (Dong et al. 2023; Xu et al. 2024). In contrast, the extracellular proteome captures processes at the cell–environment interface. It may include secreted and surface-associated proteins, extracellular enzymes, metal-binding proteins, and proteins associated with extracellular vesicles or polymeric matrices. It is of particular interest that some extracellular enzymes identified in secretome analysis under stimulating conditions (i.e., PTE-induced stress) can be produced even on a large scale if their activity could be directly employed in bioremediation procedures, as well as metal binding, sequestration, immobilization, or transformation, often in association with EPS and biofilm-related strategies (He et al. 2023; Munir Ahamed et al. 2024; Sarasa-Buisan et al. 2024). Zhang et al. (2023) performed comparative secretomics and cellular proteomics in PTE-tolerant *Vibrio cholerae* isolates, linking intracellular and extracellular responses to membrane biophysical changes, including fluidity, permeability and hydrophobicity. Their data showed that proteome-level changes can support biochemical phenotypes, highlight shared defense modules such as RND/ABC efflux systems, oxidative-stress enzymes, and EPS-associated extracellular responses, and resolve metal-specific differences across stresses (Zhang et al. 2023).

Representative case studies illustrate how proteomics can help interpret intracellular tolerance mechanisms. Comparative proteomic analysis in *Pannonibacter phragmitetus* BB linked sequential Cr(VI)/Pb(II) bioremoval to pathways involved in Cr(VI) reduction, Pb(II) adsorption, precipitation and siderophore-associated complexation, with oxidative stress control and ion homeostasis (Liao et al. 2020).

Similarly, Cr(VI) exposure in both *Bacillus toyonensis* SFC 500-1E and *Shewanella oneidensis* MR-1 was associated with broad metabolic rewiring and detoxification-related modules, as shown by variation in proteome profiles of many transporters, thioredoxins, chaperones, and other stress-response proteins. In particular, in *B. toyonensis* SFC 500-1E, Fernandez et al. (2023) combined Gel-LC with gel-free shotgun nanoUHPLC-ESI-MS/MS, identifying 400 differentially expressed proteins across Cr(VI) and Cr(VI)+phenol conditions, including ABC transporters, an iron-siderophore transporter, metal-binding transcriptional regulators and proteins involved in thioredoxin-, SOS- and chaperone-mediated stress responses (Fernandez et al. 2023). In *S. oneidensis* MR-1, Gang et al. (2019) used TMT-based quantitative proteomics to quantify approximately 2500 proteins and reported coordinated pathway-level changes consistent with chromium stress adaptation (Gang et al. 2019).

Fungal proteomics presents additional challenges compared with many bacterial systems. Protein extraction can be complicated by a rigid, heterogeneous cell wall, filamentous growth, extracellular polysaccharides, and, in some species, melanized or highly recalcitrant structures (Ning et al. 2021). Moreover, the limited availability of well-annotated reference proteomes for environmental and non-model fungi can reduce protein identification rates and leave many detected proteins annotated as hypothetical or uncharacterized (Bouqellah et al. 2023). These issues are particularly relevant for secretomic studies, where the extracellular protein fraction may include both actively secreted proteins and intracellular proteins released through cell lysis or stress-induced leakage; therefore, fungal proteomic data require careful interpretation, especially when linking extracellular proteins to metal binding, transformation or immobilization processes (Scott et al. 2024). Despite these limitations, fungal proteomics has revealed both common and metal-specific responses. In *Oidiodendron maius*, Chiapello et al. (2015) combined 2D-GEL and shotgun MS-based proteomics to distinguish common and metal-specific responses under Cd and Zn stress. Both metals induced Cu/Zn superoxide dismutase and ubiquitin, while the presence of agmatinase suggested a possible role for polyamine biosynthesis in fungal metal adaptation. Cd specifically enriched Hsp90-family chaperones, cytoskeletal proteins and translation-related components, whereas Zn mainly upregulated energy and carbohydrate metabolism (Chiapello et al. 2015). For a more application-oriented metal removal phenotype, *Pleurotus ostreatus* ISS-1 was investigated by iTRAQ quantitative proteomics alongside physicochemical assays, including FTIR and XRD. The integrated evidence supported a multimodal Pb-removal mechanism involving biosorption, bioaccumulation and precipitation, while implicating ABC transporters, cytochrome P450, peroxisome-related functions and calcium signaling in tolerance and detoxification (Wang et al. 2019). Likewise, Zeiner et al. (2016) combined LC-MS/MS secretomics with genomic and bioinformatic annotation in Mn(II)-oxidizing ascomycetes and reported secretomes enriched in carbohydrate-active enzymes and redox-active auxiliary activity enzymes, including AA3 GMC oxidoreductases and AA1 multicopper oxidases, such as laccase-like proteins. This profile is consistent with extracellular redox chemistry and metal/mineral interactions involved in biotransformation and immobilization processes (Zeiner et al. 2016). A subsequent functional study demonstrated protein-dependent Mn(II) oxidation in cell-free fungal secretomes and identified both copper- and FAD-dependent oxidation mechanisms, with tyrosinase, bilirubin oxidase, glyoxal oxidase and GMC oxidoreductases among the candidate Mn(II)-oxidizing enzymes (Zeiner et al. 2021). Table 4 summarizes the major functional classes of protein-level responses associated with microbial adaptation and PTE bioremediation.

**Table 4:** Major functional classes of proteins and protein systems associated with microbial adaptation and bioremediation of potentially toxic elements (PTEs)

Functional class	Main function	Representative proteins, systems or pathways	Organism group	Key references
Metal sensing, transport and homeostasis	Regulation of metal availability, intracellular concentration and export	ABC and RND transporters; P-type ATPases; metal-binding regulators; iron-siderophore transporters	Bacteria and fungi	(Liao et al. 2020; Fernandez et al. 2023; Zhang et al. 2023)
Metal transformation and redox processes	Enzymatic transformation of PTEs and regulation of cellular redox chemistry	Reductases; oxidoreductases; cytochromes; multicopper oxidases; GMC oxidoreductases	Bacteria and fungi	(Zeiner et al. 2016, 2021; Gang et al. 2019; Liao et al. 2020)
Oxidative stress and antioxidant defense	Protection against ROS and maintenance of redox balance	SOD; thioredoxin systems; glutathione-related proteins; peroxidases	Bacteria and fungi	(Chiapello et al. 2015; Fernandez et al. 2023; Zhang et al. 2023)
Metal binding, sequestration and immobilization	Reduction of PTE bioavailability through binding, adsorption, sequestration and precipitation	Metal-binding proteins; siderophore-associated proteins; calcium-associated processes; EPS-associated proteins	Bacteria and fungi	(Wang et al. 2019; Liao et al. 2020; Sarasa-Buisan et al. 2024)
Extracellular and secreted metal-interaction systems	Metal transformation and immobilization at the cell–environment interface	Laccase-like proteins; tyrosinase; bilirubin oxidase; glyoxal oxidase; GMC oxidoreductases; extracellular enzymes	Bacteria and fungi	(Zeiner et al. 2016, 2021; He et al. 2023; Sarasa-Buisan et al. 2024)
Stress response, protein homeostasis and DNA protection	Maintenance of protein and genome integrity during PTE exposure	Hsp90; chaperones; ubiquitin; SOS-response and DNA-repair proteins	Bacteria and fungi	(Chiapello et al. 2015; Gang et al. 2019; Fernandez et al. 2023)
Metabolic reprogramming	Redistribution of energy and biosynthetic metabolism to sustain PTE adaptation	Carbohydrate and energy metabolism; TCA-associated pathways; protein synthesis and turnover	Bacteria and fungi	(Chiapello et al. 2015; Gang et al. 2019; Fernandez et al. 2023)
Membrane and cell-envelope adaptation	Adjustment of membrane properties and cell-surface interactions under PTE stress	Membrane proteins; transport systems; EPS-associated proteins; proteins affecting membrane fluidity and permeability	Bacteria and fungi	(Wang et al. 2019; Zhang et al. 2023)

The table summarizes the main functional classes of proteins and protein systems identified through proteomic analyses of microorganisms exposed to potentially toxic elements (PTEs). Representative proteins, systems or pathways, the microbial groups in which they have been reported, and key supporting references are provided. The classification encompasses both bacterial and fungal responses. It highlights the complementary information provided by cell-associated and extracellular proteomes in identifying mechanisms potentially involved in PTE tolerance, transformation, immobilization and bioremediation

Abbreviations: *ABC* ATP-binding cassette, *RND* resistance–nodulation–division, *ROS* reactive oxygen species, *SOD* superoxide dismutase, *GMC* glucose–methanol–choline, *EPS* extracellular polymeric substances, *Hsp90* heat shock protein 90, *TCA* tricarboxylic acid cycle

### Metabolomics as painter of biochemical outcome

Metabolomics can provide a biochemical readout of microbial responses to PTE exposure by profiling low-molecular-weight compounds, generally in the range of approximately 50–1500 Da, that are produced, accumulated, consumed or released during metabolic activity (Goff et al. 2023; Tian et al. 2024). Compared with genomics, transcriptomics and proteomics, metabolomics is closer to the biochemical phenotype because it captures metabolic outputs that may directly influence metal mobility, bioavailability or toxicity (Goff et al. 2023). In PTE bioremediation studies, this information is particularly relevant because microbial metabolites can contribute to metal chelation, redox transformation, pH modulation, precipitation, biosorption, mobilization or immobilization. However, metabolomic changes should not be interpreted automatically as evidence of bioremediation, since they may also reflect general stress responses or metabolic impairment caused by metal toxicity (Tian et al. 2024; Reyes-Sánchez et al. 2025). The complexity of microbial metabolome requires the use of multiple analytical platforms for comprehensive detection as Nuclear Magnetic Resonance (NMR) Spectroscopy, Capillary Electrophoresis-Mass Spectrometry (CE-MS), Fourier Transform Infrared (FTIR), ion mobility mass spectrometry (IMS-MS), Gas Chromatography-Mass Spectrometry (GC-MS) and Liquid Chromatography-Tandem Mass Spectrometry (LC-MS/MS) (Naumann et al. 2023; Jeppesen and Powers 2023), according to their concentration, their stability and their physical-chemical properties, such as their volatility. Among these analytical techniques, LC-MS/MS is particularly valuable in this field due to its sensitivity, versatility, and suitability for both targeted and untargeted analyses (Goff et al. 2023; Tian et al. 2024; Reyes-Sánchez et al. 2025).

This approach enables detection of metabolite classes potentially involved in metal interaction and stress adaptation, including metallophores, extracellular polymeric substances (EPS)-associated metabolites, organic acids, and biosurfactants (Gao and Xu 2015; Hajnajafi and Iqbal 2025) (see Table 5 for a summary). Because microbial metabolites are structurally diverse, organizing and interpreting untargeted LC-MS/MS data can be particularly challenging and requires dedicated data-mining strategies. Molecular networking organizes MS/MS spectra by spectral similarity. It compares them with reference fragmentation patterns in public libraries such as GNPS, facilitating annotation of structurally related metabolites and exploration of complex microbial metabolomes (Quinn et al. 2017).

Among the metabolite classes relevant to PTE bioremediation, metallophores, including siderophores, represent a key molecular class because of their ability to coordinate metal ions (Skeba et al. 2023; Calderón Celis et al. 2023). These small- to medium-sized molecules feature multiple oxygen- and, in some cases, nitrogen-containing functional groups, including hydroxamate, catecholate and carboxylate moieties, which enable metal binding and can influence their ionization behavior during MS-based analyses (Xu et al. 2023). Although siderophores are primarily associated with Fe acquisition, they can also interact with other metals, including Cu(II), Zn(II), Cd(II), Pb(II), Ni(II) and Ga(III), thereby contributing to metal tolerance/bioremediation and in some cases recovery (Wu et al. 2023; Xu et al. 2023; Skeba et al. 2023). In this context, genome mining can predict the biosynthetic potential for metallophore production. In contrast, metabolomics can provide direct evidence of their production by detecting apo- and holo-metallophore species in complex extracts (Wu et al. 2023).

In bacteria, metabolomic and metabolite-profiling approaches have been particularly useful for identifying extracellular molecules involved in metal binding, mobilization, reduction or recovery. Representative studies illustrate this potential. Baars et al. (2018) identified the petrobactin-related siderophores rhodopetrobactin A and B in the supernatant of *Rhodopseudomonas palustris* using a combination of HRMS, UV/Vis spectroscopy and NMR (Baars et al. 2018). The analysis of metal stable-isotope fingerprints of metabolites bound to Fe(III) and MoO₂²⁺ also enabled the identification of 3,4-dihydroxybenzoic acid (3,4-DHBA), indicating the presence of a catechol moiety and supporting the discrimination of siderophore-like compounds within a complex metabolome. Similarly, Zytnick et al. (2024) targeted hydroxamate-containing metabolites using LC-MS/MS in both positive and negative ionization modes, together with molecular networking and NMR, to characterize methylolanthanin and rhodopetrobactin B in *Methylobacterium extorquens* AM1. These compounds were associated with lanthanide binding and recovery, with *M. extorquens* accumulating approximately 13.3 mg of Nd from Nd₂O₃ per gram of dry biomass, highlighting its potential for the selective recovery of lanthanides from electronic waste and permanent magnets (Zytnick et al. 2024).

Other bacterial studies further support the ecological and applicative relevance of siderophore-mediated metal interactions. Monych and Turner (2020) showed that pyochelin and its precursor dihydroaeruginoic acid, produced by *Pseudomonas aeruginosa*, increase tolerance to Cu and Ag, suggesting that siderophore-related metabolites can contribute to metal stress adaptation beyond Fe acquisition (Monych and Turner 2020). In rhizosphere-associated systems, siderophore-producing bacteria may also support phyto-microbial remediation. For instance, UHPLC-QTOF/MS analysis identified hydroxamate-, catecholate- and phenolate-type siderophores in *Streptomyces* isolates from metal-polluted rhizospheres, with the ability to chelate Cd(II) (Złoch et al. 2016). More recently, large-scale environmental detection of metallophores by two-dimensional solid-phase extraction liquid chromatography–mass spectrometry (2D-SPE-LC-MS) revealed 53 compounds in peatlands, supporting their role in Fe and Cu scavenging in native soils (Calderón et al. 2023). Besides siderophores, extracellular polymeric substances (EPS) and EPS-associated low-molecular-weight components also show strong potential to contribute to PTE bioremediation. EPS are heterogeneous polymeric matrices composed of polysaccharides, proteins, lipids, nucleic acids and associated small molecules. They can contribute to metal binding, trapping, immobilization, or chemical transformation, thereby reducing metal bioavailability and toxicity. However, their polymeric and heterogeneous nature complicates direct analytical characterization. In this context, metabolomic approaches can help identify soluble ligands and low-molecular-weight components associated with metal binding, reduction or complexation (Li and Yu 2014; Luo et al. 2022). For example, enhanced EPS production by *Bacillus cereus* KMS3-1 under Pb, Cu and Cd exposure was associated with improved metal adsorption and detoxification capacity (Mathivanan et al. 2021). Luo et al. (2022) showed that EPS from *Staphylococcus saprophyticus* promoted Cr(VI) reduction, reaching 48.85% efficiency with EPS alone and 99.4% when EPS was combined with tryptophan. LC-MS/MS analysis suggested that tryptophan-like components were oxidized into aromatic compounds that acted as biochemical protective agents and contributed to the transformation of toxic Cr(VI) into the more stable and less toxic Cr(III) form (Luo et al. 2022).

Biosurfactants represent another relevant class of metabolites involved in PTE remediation. These amphiphilic molecules, produced by microorganisms such as *Pseudomonas*, *Bacillus*, *Candida*, and *Starmerella bombicola*, can complex metal ions, promote their desorption from solid matrices, and enhance removal through mobilization, precipitation, or micellar solubilization (Mishra et al. 2021; Pardhi et al. 2022). In particular, anionic biosurfactants such as rhamnolipids and surfactins interact efficiently with divalent and trivalent cations, increasing metal transfer into the aqueous phase and facilitating recovery or removal (Mosa et al. 2016; Lopes et al. 2021; Silva et al. 2022). Consistently, untargeted LC-MS metabolomics of *Pseudomonas monteilii* X1 under Cd and Zn stress revealed changes in metabolites associated with biosurfactant precursor pathways, supporting their contribution to metal biosorption (Wang et al. 2024b).

Microbially produced organic acids also contribute to PTE remediation by chelating metals, altering pH, and increasing metal mobility and bioavailability, particularly in bioleaching processes. Molecules such as oxalic and citric acid can form complexes with various metals (e.g., Al, Fe, Mg, Ca), and the strength of these complexes significantly affects the leaching process efficiency (Sarkodie et al. 2022). Because of their high abundance and strong ionization in negative mode, LC-MS/MS is one of the most suitable ways to detect, identify, and quantify these molecules. These studies confirm that metabolomics can be a powerful tool in PTE bioremediation research and in applied settings, particularly for linking molecular responses to measurable process outputs (Sarkodie et al. 2022). In fungi, the application of metabolomics to PTE bioremediation remains limited by the kingdom’s high biological and metabolic diversity. Fungal responses to metal exposure can vary substantially among species, growth forms and experimental conditions, and often involve multiple interconnected levels, including extracellular metabolite release, cell-wall interactions, redox balance, osmotic stress protection and intracellular detoxification (Seena et al. 2020; Liu et al. 2022). In addition, many environmental and non-model fungi remain poorly characterized in terms of genome annotation, metabolic pathways and reference metabolite information, making the identification and validation of specific metabolite targets more challenging than in many bacterial systems (Gil-de-la-Fuente et al. 2021; Zhang et al. 2022a). This complexity partly explains why, although fungi are expected to produce metabolites complementary to those observed in bacteria, including organic acids, soluble ligands, siderophore-like compounds, thiol-containing molecules, polyols and other stress-related metabolites, fungal metabolomics is less frequently applied as a comprehensive untargeted approach (Seena et al. 2020; Liu et al. 2022; Wang et al. 2023; Singh et al. 2025). Nevertheless, the available studies show that fungal metabolomics can provide detailed information on the molecular responses associated with PTE exposure and tolerance. In *Neonectria lugdunensis* exposed to mine drainage waters, lipid and amino-acid profiling revealed isolate-dependent changes, particularly in triacylglycerols, lysophosphatidylcholines and amino-acid composition (Seena et al. 2020). In *Aspergillus niger* exposed to Cu, combined metabolomic and secretomic analyses associated mannitol with fungal resistance and gluconic acid with Cu desorption through chelation (Perdigão Cota De Almeida et al. 2021). Integrated transcriptomic and metabolomic analysis of *Trametes pubescens* under Cd stress revealed a reorganization of amino-acid and nitrogen metabolism toward α-ketoglutarate and the TCA cycle, supporting ATP and NADPH production under oxidative stress (Liu et al. 2022). Similarly, untargeted metabolomics of *Paraisaria dubia* exposed to Zn identified changes in amino acids, nucleosides, organic acids, polyols, betaine and choline, which were associated with osmotic regulation, antioxidant protection and stress adaptation (Wang et al. 2023). In a *Penicillium* strain exposed to U(VI), non-targeted metabolomics revealed alterations in organic-acid, amino-acid, TCA-cycle and nucleotide metabolism. At the same time, complementary analyses indicated uranium association with functional groups and mineralized deposits at the fungal surface (Zhang et al. 2022a). Alongside these metabolomics-based studies, the literature often relies on metabolite-based functional assays, targeted organic-acid profiling, culture-supernatant chemistry and fungal bioleaching experiments. These approaches do not reconstruct the whole metabolome. However, they can provide mechanistic information by linking extracellular metabolite production, pH changes and metal concentrations in the culture supernatant or leachate to measurable processes such as metal mobilization, complexation, precipitation or immobilization (Nkuna and Matambo 2024; Li et al. 2024b). In this context, fungal bioleaching and biorecovery studies are particularly informative because they assess fungi’s ability to mobilize metals from solid matrices through extracellular chemistry (Dusengemungu et al. 2021; Trivedi et al. 2022). *Aspergillus* and *Penicillium* species have been frequently discussed for their ability to release organic acids and soluble ligands that acidify the medium and promote metal solubilization through acidolysis and complexation (Dusengemungu et al. 2021). Recent overviews of fungal bioleaching emphasize that, unlike bacterial bioleaching processes mainly based on iron- and sulfur-oxidizing microorganisms, fungal metal mobilization is largely associated with organic-acid production, chelation, and culture-supernatant chemistry (Dusengemungu et al. 2021; Rendón-Castrillón et al. 2023; Tezyapar Kara et al. 2023). Similarly, fungal biorecovery from e-waste has been described as a process in which fungal metabolism and extracellular compounds may contribute to mobilizing and recovering valuable metals from complex solid residues (Trivedi et al. 2022).

Therefore, fungal metabolomics and process-oriented metabolite studies provide complementary information. Metabolomic approaches can reveal coordinated intracellular and extracellular responses and identify candidate metabolites and pathways associated with metal tolerance. In contrast, targeted assays and bioleaching experiments can directly evaluate how specific extracellular products influence metal mobilization, complexation or immobilization. Their interpretation should therefore be integrated with metal speciation, mass-balance or removal-efficiency measurements, recovery yield and toxicity assays (Liang and Gadd 2017; Liu et al. 2022; Trivedi et al. 2022; Doydora et al. 2024).

**Table 5:** Major functional classes of metabolites associated with microbial adaptation and bioremediation of potentially toxic elements (PTEs)

Functional class	Main function	Representative metabolites, compounds or pathways	Organism group	Key references
Metallophores and siderophores	Metal chelation, metal acquisition, tolerance and mobilization; potential contribution to metal recovery	Hydroxamate-, catecholate- and carboxylate-containing siderophores; petrobactin-related compounds; rhodopetrobactin A/B; methylolanthanin; pyochelin	Mainly bacteria, also fungi	(Złoch et al. 2016; Baars et al. 2018; Monych and Turner 2020; Skeba et al. 2023; Calderón et al. 2023; Zytnick et al. 2024)
Organic acids and soluble metal-chelating metabolites	Metal complexation, pH modulation, solubilization and mobilization; particularly relevant to bioleaching	Citric acid; oxalic acid; gluconic acid; other carboxylic acids and soluble organic ligands	Bacteria and fungi	(Perdigão et al. 2021; Dusengemungu et al. 2021; Trivedi et al. 2022; Sarkodie et al. 2022)
Extracellular polymeric substance (EPS)-associated metabolites and soluble ligands	Metal binding, adsorption, trapping, immobilization and chemical transformation at the cell–environment interface	Tryptophan-related compounds; soluble EPS components; low-molecular-weight ligands associated with EPS matrices	Mainly bacteria, also fungi	(Li and Yu 2014; Mathivanan et al. 2021; Luo et al. 2022)
Biosurfactants and amphiphilic metabolites	Metal complexation, desorption, mobilization, micellar solubilization and enhanced metal transfer to the aqueous phase	Rhamnolipids; surfactins; lipopeptides; glycolipids	Bacteria and fungi	(Mosa et al. 2016; Lopes et al. 2021; Mishra et al. 2021; Silva et al. 2022; Pardhi et al. 2022; Wang et al. 2024b)
Thiol-containing and redox-active metabolites	Metal binding, intracellular detoxification and maintenance of redox balance under PTE-induced oxidative stress	Glutathione and related thiol-containing compounds; other redox-active metabolites	Bacteria and fungi	(Seena et al. 2020; Liu et al. 2022; Wang et al. 2023)
Amino acids and nitrogen-containing metabolites	Osmotic regulation, metal complexation, antioxidant protection and metabolic adaptation	Amino acids; histidine-related compounds; arginine-related metabolites; choline; betaine	Bacteria and fungi	(Seena et al. 2020; Huang et al. 2022; Liu et al. 2022; Wang et al. 2023)
Polyols and osmoprotective metabolites	Osmotic protection, cellular stabilization and adaptation to metal-induced stress	Mannitol; polyols; compatible solutes	Mainly fungi, also bacteria	(Perdigão et al. 2021; Wang et al. 2023)
Central carbon and energy metabolites	Metabolic reprogramming and provision of ATP, reducing equivalents and biosynthetic precursors during PTE stress	TCA-cycle metabolites; glycolytic intermediates; pentose phosphate-related metabolites; organic acids	Bacteria and fungi	(Zhang et al. 2022a; Liu et al. 2022; Wang et al. 2023)
Lipid and membrane-associated metabolites	Adaptation of membrane composition and cellular integrity under PTE stress	Triacylglycerols; lysophosphatidylcholines; other membrane-associated lipids	Mainly fungi	(Seena et al. 2020)
Nucleosides and nucleotide-related metabolites	Regulation of energy metabolism, nucleotide turnover and cellular stress responses	Nucleosides; nucleotide-related metabolites	Mainly fungi	(Zhang et al. 2022a; Wang et al. 2023)
Extracellular redox-active and metal-transforming metabolites	Reduction or oxidation of PTEs and modification of metal speciation and mobility	Phenolic compounds; tryptophan-derived metabolites; redox-active aromatic compounds	Bacteria and fungi	(Perdigão et al. 2021; Zeiner et al. 2021; Luo et al. 2022)
Secondary metabolites involved in metal stress adaptation	Chemical defense, signaling, chelation and modulation of microbial interactions under PTE exposure	Diverse secondary metabolites detected through untargeted metabolomics and molecular networking	Bacteria and fungi	(Quinn et al. 2017; Calderón et al. 2023; Reyes-Sánchez et al. 2025)

The table summarizes the major classes of microbial metabolites and biochemical pathways associated with adaptation to and bioremediation of PTEs. Representative metabolites or metabolic pathways, the microbial groups in which they have been described, and key supporting references are provided. These metabolite classes may contribute to PTE adaptation through metal chelation, redox transformation, pH modulation, biosorption, mobilization, sequestration, precipitation, or immobilization. The table encompasses both bacterial and fungal systems and highlights the complementary role of metabolomics in linking molecular responses to biochemical processes and measurable PTE-remediation outcomes

Abbreviations: *EPS* extracellular polymeric substances, *ATP* adenosine triphosphate, *TCA* tricarboxylic acid cycle

## Conclusions: strengths, weaknesses and perspectives

In the so-called post-genomic era, the flourishing of new technologies accompanied by the development of software capable of managing enormous amounts of data (high-throughput) has allowed the various omics approaches, of which the main but not exhaustive are genomics, transcriptomics, proteomics and metabolomics, to rapidly establish themselves in the attempt to decipher biological processes in their maximum completeness and complexity. Although their primary area of application has been humans and a few animal models, in the last decade, in light of the increasingly clear potential of these applications, omics sciences are finding their place in deciphering biological processes of various origins, including those related to niche organisms/microorganisms adaptation, such as those potentially exploitable in bioremediation applications. Omics technologies have accelerated research into the bioremediation of microbial PTEs by shifting the focus from descriptive observations toward mechanism-informed models of tolerance, transformation, sequestration, and removal. However, the practical application of omics to investigate microorganisms’ adaptation mechanisms remains constrained by incomplete reference genomes, limited annotation of non-model taxa, and database bias, particularly for environmental isolates and complex consortia. Environmental matrix complexity affects all omics layers, but its impact becomes increasingly critical and less easily standardized when moving from nucleic acids to proteins and metabolites; genomic and transcriptomic approaches are generally more widely adopted in bioremediation studies because DNA- and RNA-based workflows are more technically mature, scalable and supported by relatively standardized protocols for extraction, library preparation, sequencing and bioinformatic analysis, while proteomics and metabolomics are often more strongly influenced by the physicochemical complexity of the sample, as humic substances, salts, metals and organic or inorganic contaminants can interfere with protein/metabolite extraction, digestion and separation, ionization and consequent molecular identification (Arıkan and Muth 2023; Thukral et al. 2023; Nebauer et al. 2024) (as summarized in Table 1).

In proteomics, a further major bottleneck is the dependence on high-quality protein/gene sequence databases, since reliable protein identification requires an appropriate reference proteome, usually derived from a well-assembled and accurately annotated genome or metagenome, something particularly challenging for environmental isolates where genomic information is often incomplete and many predicted proteins remain annotated as hypothetical or uncharacterized (Arıkan and Muth 2023; Nebauer et al. 2024). Metabolomics partly bypasses the need for a reference genome but introduces different analytical and interpretative bottlenecks. In untargeted workflows, metabolite annotation and identification remain major challenges because spectral libraries and chemical databases are still incomplete compared with the vast diversity of metabolites produced by each microbial category (Thukral et al. 2023). In addition, although exometabolomics can be particularly informative because extracellular metabolites directly interact with and influence PTEs, data interpretation is further complicated by the possible presence of abiotic metal–metabolite interactions, degradation products, and metabolites transformed or consumed by other organisms, which ultimately hinder the attribution of specific signals to a defined microbial source or function (Douglas 2020; Thukral et al. 2023). Despite these limitations, integrating multiple omics layers remains essential to obtain a more comprehensive and mechanistically grounded view of microbial responses to PTE exposure. By combining genomic, transcriptomic, proteomic, and metabolomic information, it is possible to move from what an organism or microbial community is genetically able to do to what and how it can adapt to a particularly hostile environment. This is possible by exploring transcriptionally activated genes and, finally, identifying proteins and metabolites produced, accumulated, or released under specific PTE-stress conditions (Sharma et al. 2022; Arıkan and Muth 2023).

More broadly, integrating omics data with phenotypic assays is crucial to translate molecular information into biotechnological relevance. Functional assays, such as growth analysis, metal tolerance tests, removal efficiency measurements, biosorption and bioaccumulation assays, metal speciation or toxicity reduction tests, provide direct evidence of what a microorganism or consortium can perform under defined conditions. Omics approaches, in turn, can link these observed outcomes to the underlying molecular mechanisms, helping to clarify whether PTE tolerance, removal, or immobilization evidence is associated with mechanisms such as efflux systems, bioaccumulation or biosorption, extracellular binding, biomineralization, redox transformation, chelation or broader metabolic adaptation (Sharma et al. 2022). Moreover, understanding these mechanisms can optimize process conditions, including PTE concentration, exposure time, pH, salinity, nutrient availability, and the use of biofilms or identified extracellular products. In this sense, omics can support the rational design of microbial consortia by identifying bacteria, fungi, and other organisms that use different but potentially complementary remediation strategies (Efremenko et al. 2024). Bacterial–fungal consortia may combine the extensive metal-binding surfaces, extracellular ligands, organic-acid production, and biomineralization potential often associated with fungal biomass with bacterial mechanisms such as EPS and siderophore production, redox transformations, sulfate reduction, and metal precipitation. Fungal hyphae may also provide a spatial scaffold that supports bacterial attachment, dispersal, and biofilm organization (Ameen 2023; Efremenko et al. 2024). Omics characterization of individual isolates can therefore provide a mechanistic basis for selecting microorganisms with potentially complementary traits and for defining molecular markers to monitor their persistence and activity. However, functions detected in monoculture may not remain unchanged after consortium assembly, because nutrient competition, metabolite exchange, changes in pH and redox conditions, or the production of inhibitory compounds may generate synergistic, neutral, or antagonistic outcomes (Efremenko et al. 2024; Li et al. 2024a). Candidate combinations should therefore be tested experimentally, first in controlled co-cultures and subsequently in microcosms reproducing the relevant environmental matrix and geochemical conditions, to determine whether the selected members remain active and improve PTE removal, immobilization, detoxification, or reduction of bioavailability (Ameen 2023; Efremenko et al. 2024; Li et al. 2024a). In a bioremediation protocol set up, the monitoring phase is addressed to the identification of marker genes, transcripts, proteins or metabolites which can help to determine which organism is associated with a given observed function or remediation pathway and at the same time support the evaluation of potential limitations and ecological risks, such as the mobilisation of undesirable PTEs, which in a real environmental application may represent an additional hazard rather than a remediation outcome (Arıkan and Muth 2023; Li et al. 2023). Table 6 summarises a possible workflow for combining omics layers across the phases of a real bioremediation project (screening, implementation, monitoring) and identifies which combinations are realistically feasible in the field versus the laboratory.

**Table 6:** Proposed tiered omics strategy for bioremediation across the main phases of an operational workflow

Phase	Main objective	Recommended omics combinations	Outcome
1. Screening	Identify candidate strains, taxa, pathways, and mechanisms	Genomics/metagenomics → transcriptomics/metatranscriptomics → selective proteomics, secretomics or metabolomics	Link functions to specific microorganisms and confirm pathway activation under PTE exposure (Ayala-Muñoz et al. 2020; Li et al. 2023)
2. Implementation	Optimize treatment conditions and consortia	Comparative metagenomics + transcriptomics/proteomics/metabolomics	Identify active community members, optimal environmental conditions, limiting responses, and molecular markers for field monitoring (Goff et al. 2023; Arıkan and Muth 2023; Galea et al. 2024)
3. Monitoring	Verify remediation performance and ecological stability	Targeted qPCR/RT-qPCR, protein or metabolite assays, complemented periodically by amplicon sequencing, metagenomics or metatranscriptomics; use metaproteomics/metabolomics selectively	Track validated molecular signatures while linking them to chemical, geochemical, and phenotypic indicators of remediation. Transcript-based markers should be interpreted cautiously because pathway expression does not necessarily correspond directly to remediation rates (Madison et al. 2023; Vogel et al. 2023)

The table summarizes a possible strategy for integrating different omics layers across the screening, implementation, and monitoring phases of a bioremediation project, highlighting the most appropriate combinations for each phase and their potential contribution to evaluating remediation performance

Abbreviations: *qPCR* quantitative polymerase chain reaction, *RT-qPCR* reverse transcription quantitative polymerase chain reaction

The choice and depth of omics should match the available cost, turnaround time, sample complexity, infrastructure, and analytical expertise. Consequently, laboratory studies can support broader multi-omics combinations. In contrast, large-scale field applications are generally better suited to targeted assays and lower-cost community profiling, supplemented by periodic untargeted analyses. This tiered design maintains mechanistic resolution while keeping routine monitoring feasible at the spatial and temporal scales of real bioremediation projects (Habibi et al. 2023; Arıkan and Muth 2023; Davis et al. 2025). In parallel, the “emerging analytical technologies” (long-read, single-cell, spatial omics, ion mobility), integrated with increasing uptake of AI and machine learning, may further improve the outcome quality and the integration of heterogeneous omics and geochemical datasets, helping to identify robust cross-site patterns and to link molecular signatures with process-level outcomes (Blessing and Olateru 2025). In this perspective, the integration of omics sciences into environmental biotechnology can support the development of more reliable, scalable, and site-relevant bioremediation strategies, ultimately contributing to the mitigation of PTE contamination and its impacts on ecosystems and human health (Sharma et al. 2022; Arıkan and Muth 2023; Blessing and Olateru 2025).

## Data Availability

No datasets were generated or analysed during the current study.
